# Data on germination, growth and morphological changes of oil palm (*Elaeis guineensis* Jacq.) zygotic embryos during *in vitro* culturing

**DOI:** 10.1016/j.dib.2019.104975

**Published:** 2019-12-16

**Authors:** D.S. Sparjanbabu, H.S. Prasanna, D. Ramajayam, P. Naveenkumar, M.S.R. Krishna, B. Susanthi

**Affiliations:** a Department of Biotechnology, KL Deemed to be University, Vaddeswaram, 522 502, A.P, India; b Division of Crop Improvement, ICAR-Indian Institute of Oil Palm Research, Pedavegi, 534 450, A.P, India; c Horticultural College and Research Institute, Dr. Y.S. R. Horticulture University, Venkataramannagudem, 534 101, A.P, India; d Division of Crop Improvement, ICAR-National Research Centre for Banana, Tiruchirapally, 620 102, Tamil Nadu, India; e Division of Crop Improvement, ICAR-Directorate of Floricultural Research, Pune, 411 005, Maharashtra, India

**Keywords:** Oil palm, Zygotic embryo, *in vitro*, Genotype, Germination index

## Abstract

Oil palm (*Elaeis guineensis* Jacq.) from being almost unknown crop a mere three decades ago is now the most consumed and the most traded edible oil in the world. It is a highest yielding crop producing on an average 4 to 6 tons of oil per ha per year. Due to its innumerable uses in the food, oleochemicals and biofuel industries, cultivation of oil palm has expanded enormously in recent years. Since oil palm is a perennial monocotyledonous species with a single growing apex, the plant cannot be multiplied vegetatively and the conventional propagation through seed is limited by dormancy. Thus *in vitro* germination has become the key method for multiplication of elite oil palm genotypes. Although there are several reports on *in vitro* germination of oil palm, still there is a lack of an efficient & repeatable method. Hence an attempt is made to standardize the suitable culture media for direct germination from mature oil palm zygotic embryos. The data presented here represents the effect of genotypes, pretreatments and culture media on Mean Germination Time, Speed of Germination Index, Shoot Formation Index and Root Formation Index during *in vitro* culturing of oil palm zygotic embryos.

**Table:** Specifications Table

Subject area	Agricultural Sciences
More specific subject area	Plant Tissue Culture
Type of data	Table
How data was acquired	By the manual observation of different embryo germination stages and plant growth & development stages
Data format	Raw and analyzed
Experimental factors	The experimental design was three factorial treatment combinations of genotype, pretreatment and culture media arranged in a randomized complete block design.
Experimental features	The whole set of experiment was repeated twice with 25 embryos per treatment per replication. Determination of mean germination time, the speed of germination index, the speed of shoot formation index and the speed of root formation index taken by oil palm zygotic embryos.
Data source location	Tissue Culture Laboratory, Division of Crop Improvement, ICAR-Indian Institute of Oil Palm Research, Pedavegi, West Godavari District, Andhra Pradesh, India.
Data accessibility	Analyzed tabulated data is with this article. Statistical analysis data and raw data are added as supplementary files.
Related research article	Sparjanbabu et al. [1] Effect of culture media, plant growth regulators and genotypes on growth and developmental stages of oil palm (*Elaeis guineensis* Jacq.) zygotic embryos. Indian Journal of Agricultural Research.,53 (2) 2019:143–150.

**Table:** 

**Value of the Data** - The present data gives details on the effect of treatment combinations on *in vitro* germination of the oil palm zygotic embryos. - Data provides the genotypic effect on *in vitro* germination of oil palm zygotic embryos, which will be useful to evaluate and select the advanced breeding material and hybrid seed production. - Data obtained by the factorial experiment allows understanding the effect of each factor on the response variable as well as the effect of interactions between factors on the responsive variable. - The data presented here will be valuable for evaluating the different growth and morphological stages of zygotic embryos *in vitro*. - The data can be used as a template for the standardization of *in vitro* culture media for various genotypic specific.

## 1 Data

The present data in Table 1 shows the effect of genotype, pretreatment and culture media and their interactions on mean germination time, the speed of germination index, the speed of shoot formation index and the speed of root formation index taken by oil palm ZEs where between genotype and culture media, no significant difference was noticed for the mean germination time taken by ZEs of oil palm. With respect pretreatment, there was a significant effect. The soaked ZEs recorded more mean germination time (15.63d) than unsoaked ZEs (14.54 d). In three way interactions (between genotype, pretreatment and culture media) the mean time taken for germination of oil palm ZEs showed a significant effect (Table 1). The highest mean germination time (17.65 d) was recorded in T_16_ when compared all other treatment combinations. However, T_16_ was *on par* with T_14_, T_11_, T_3_, T_15_, T_12_, T_1_, T_13_, T_10_, T_9_ and T_2_. The lowest mean germination time (12.25 d) was recorded by T_5_ which was *on par* with T_16_, T_4_, T_7_, T_18_, T_20_, T_19_, T_7_, T_6_, T_2_ and T_9_.

**Table 1:** Effect of genotypes, pretreatments and culture media on mean germination time (MGT), speed of germination index (SGI), speed of shoot formation index (SSI) and speed of root formation index (SRI).

			Treatments	MGT	SGI	SSI	SRI
**Mean of main effects**							
G1			G1	14.62	5.63	2.11	0.21
G2			G2	15.55	5.48	1.91	0.19
	Soaked		P1	15.63	5.42	2.02	0.21
	Un soaked		P2	14.54	5.69	2.00	0.18
		MS	M1	15.14	5.09	1.52	0.00
		½ MS	M2	15.60	4.86	1.67	0.14
		MS + AC	M3	15.21	5.23	2.12	0.59
		Y3	M4	14.86	6.10	2.21	0.13
		N6	M5	14.60	6.50	2.54	0.14
**Mean of two way interactions**							
G1	Soaked		G1P1	14.68	5.78	2.13	0.22
	Un soaked		G1P2	14.55	5.48	2.09	0.21
G2	Soaked		G2P1	16.59	5.06	1.91	0.21
	Un soaked		G2P2	14.52	5.90	1.91	0.16
G1		MS	G1M1	15.30	4.89	1.50	0.00
G1		½ MS	G1M2	14.18	5.13	1.86	0.12
G1		MS + AC	G1M3	15.52	4.99	2.12	0.60
G1		Y3	G1M4	14.10	6.50	2.44	0.17
G1		N6	G1M5	13.99	6.66	2.64	0.16
G2		MS	G2M1	14.99	5.29	1.54	0.00
G2		½ MS	G2M2	17.02	4.59	1.47	0.15
G2		MS + AC	G2M3	14.91	5.48	2.12	0.57
G2		Y3	G2M4	15.63	5.71	1.98	0.10
G2		N6	G2M5	15.21	6.34	2.45	0.11
	Soaked	MS	P1M1	16.52	4.70	1.44	0.00
	Soaked	½ MS	P1M2	15.62	5.34	1.89	0.14
	Soaked	MS + AC	P1M3	16.41	4.68	1.94	0.65
	Soaked	Y3	P1M4	15.28	5.90	2.27	0.13
	Soaked	N6	P1M5	14.34	6.49	2.56	0.15
	Un soaked	MS	P2M1	13.76	5.48	1.59	0.00
	Un soaked	½ MS	P2M2	15.58	4.39	1.45	0.13
	Un soaked	MS + AC	P2M3	14.02	5.78	2.29	0.52
	Un soaked	Y3	P2M4	14.45	6.31	2.16	0.14
	Un soaked	N6	P2M5	14.87	6.51	2.52	0.13
**Mean of three way interactions**							
G1	Soaked	MS	T1 (G1P1M1)	16.17	4.87	1.50	0.00
G1	Soaked	½ MS	T2 (G1P1M2)	14.85	5.80	2.18	0.12
G1	Soaked	MS + AC	T3 (G1P1M3)	16.85	4.01	1.64	0.60
G1	Soaked	Y3	T4 (G1P1M4)	13.29	6.93	2.54	0.19
G1	Soaked	N6	T5 (G1P1M5)	12.25	7.30	2.80	0.17
G1	Un soaked	MS	T6 (G1P2M1)	14.42	4.90	1.50	0.00
G1	Un soaked	½ MS	T7 (G1P2M2)	13.51	4.46	1.54	0.13
G1	Un soaked	MS + AC	T8 (G1P2M3)	14.19	5.97	2.59	0.60
G1	Un soaked	Y3	T9 (G1P2M4)	14.90	6.07	2.35	0.14
G1	Un soaked	N6	T10 (G1P2M5)	15.74	6.01	2.47	0.15
G2	Soaked	MS	T11 (G1P2M5)	16.87	4.52	1.39	0.00
G2	Soaked	½ MS	T12 (G2P1M2)	16.39	4.88	1.59	0.17
G2	Soaked	MS + AC	T13 (G2P1M3)	15.97	5.36	2.24	0.70
G2	Soaked	Y3	T14 (G2P1M4)	17.26	4.87	2.00	0.06
G2	Soaked	N6	T15 (G2P1M5)	16.44	5.68	2.33	0.13
G2	Un soaked	MS	T16 (G2P2M1)	13.10	6.05	1.68	0.00
G2	Un soaked	½ MS	T17 (G2P2M2)	17.65	4.31	1.36	0.13
G2	Un soaked	MS + AC	T18 (G2P2M3)	13.85	5.60	2.00	0.45
G2	Un soaked	Y3	T19 (G2P2M4)	14.00	6.55	1.97	0.13
G2	Un soaked	N6	T20 (G2P2M5)	13.99	7.00	2.57	0.10
			CV (%)	11.55	18.81	21.06	62.43
			CD at 5% (G)	NS	NS	NS	NS
			CD at 5% (P)	0.91	NS	NS	NS
			CD at 5% (M)	NS	0.86	0.35	0.10
			CD at 5% (G × P)	NS	NS	NS	NS
			CD at 5% (GxM)	NS	NS	NS	NS
			CD at 5% (PxM)	NS	NS	NS	NS
			CD at 5% (GxPxM)	2.88	1.73	0.70	0.20

The speed of germination index noticed during culturing of oil palm ZEs did not showed any significant difference between genotype and pretreatments while the culture media showed a significant effect on the speed of germination index. It was faster (6.50) in the ZEs cultured in N6 media followed by Y3 media (6.10) whereas, the speed of germination index was slower (4.86) in ½ MS which was *on par* with MS + AC (5.23) and MS (5.09). In three way interactions there was a significant difference observed in speed of germination index. In 20 treatment combinations T_5_ recorded faster SGI (7.30) which was *on par* with T_20_, T_4_, T_19_, T_9_, T_16_, T_10_, T_7_, T_2_, T_15_ and T_18_ whereas the speed of germination index was lowest (4.01) in T_3_ which was *on par* with T_17_, T_7_, T_11_, T_14_, T_1_, T_12_, T_6_, T_13_, T_18_ and T_15_. The present data revealed that the growth of ZEs in 35 days in three different media tested. In the present data, swelling, expansion followed by formation of haustorium leading to the emergence of plumule from the shoot apex was shown.

The speed of shoot formation index showed no significant difference between genotype and pretreatments. The interaction effect of genotype with pretreatments, pretreatments with culture media and genotype with culture media showed non-significant effect for the speed of shoot formation index. Similarly the three way interaction effect between genotype, pretreatments and culture media was also showed non-significant.

The speed of root formation index also followed the same trend as of SSI for the effect of genotype, pretreatments, two way interaction effect (genotype with pretreatments, pretreatments with culture media and genotype with culture media) and three way interaction effect (between genotype, pretreatments and culture media).

## 2 Experimental design, materials and methods

### 2.1 Treatment details

The experimental design was three factorial treatment combinations of culture media, plant growth regulators and genotypes arranged in a randomized complete block design. The whole set of experiment was repeated twice with 20 embryos per treatment per replication.

**Table:** 

S.No.	Factors	Levels
1.	Genotypes	2 levels (*G1*and *G2*)
2.	Pretreatments	2 levels (Soaked and unsoaked)
3.	Culture media	5 levels (MS, ½ MS, MS+0.05% AC, Y3 and N6 media)

Total number of embryos inoculated = 1500 (25 test tubes per treatment x 20 treatments x 3 replications).

The material was from Dura mother palm block in ICAR-IIOPR seed garden where the material belongs to elite dura genotypes. The standardized protocol has been followed for *in vitro* germination of the zygotic embryos irrespective of genotypes. In which mature oil palm open pollinated fresh fruit bunches of four genotypes were harvested and fruitlets were depericarped using a depericarper in the seed production lab of ICAR-IIOPR, kernels obtained were surface sterilized by adding few drops of Tween-20 and then immersing the kernels in fungicide solution of (1% Carbendazim and 1% Mancozeb) and soaked in distilled water for 5 days, for attaining the required moisture content of the zygotic embryo.

Then the kernels were washed repeatedly with Tween-20 solution (10 drops/100ml v/v) for 15 minutes and washed with running tap water. They were then washed by fungicide solution (1% Carbendazim and 1% Mancozeb). They were then soaked in ethanol for 1 minute and then washed with 20% NaOCl sol for 20 minutes. Then kernels were halved and embryos were sterilized with 20% (v/v) NaOCl for 20 minutes and then washed with sterile double distilled water for three times. Three types of Basal Culture medium [[2], [3], [4]] viz., MS [5] N6 [6] & Y3 [7] were supplemented with 30g/L (W/V) Sucrose, and the pH of the medium was adjusted to 5.8 and added 8.0g/L of Agar (Clerigar™ from HIMEDIA) prior to sterilization at 121 °C for 20 min. Half MS medium was prepared by using half strength of chemicals which were used in MS medium, while MS+0.05% AC medium was prepared by adding 500 mgl^−1^ activated charcoal to the full strength MS medium.

### 2.2 Treatment combinations

After the preparation of explants and culture media, treatment imposition was carried out. The treatments consisted of three factors with 20 treatment combination (2 × 2 × 5) and 25 test tubes (25 × 150mm) per treatment *i.e.* two genotypes, two pretreatments and five culture media with three replications. Excised embryos were cultured on 10 ml medium in test tubes. All culture tubes with explants were incubated in the dark growth room at a temperature of 25±2 °C for 35 days. The numbers of germinated embryos were recorded. Embryos were considered viable when they had expanded and showed signs of Haustorium formation after 7 days of culture.

**Table:** 

S.No.	Treatments	Combinations
1.	T_1_	*G1* + Soaked + MS medium
2.	T_2_	*G1* + Soaked + ½ MS medium
3.	T_3_	*G1* + Soaked + MS + AC medium
4.	T_4_	*G1* + Soaked + Y3 medium
5.	T_5_	*G1* + Soaked + N6 medium
6.	T_6_	*G1* + Unsoaked + MS medium
7.	T_7_	*G1* + Unsoaked + ½ MS medium
8.	T_8_	*G1* + Unsoaked + MS + AC medium
9.	T_9_	*G1* + Unsoaked + Y3 medium
10.	T_10_	*G1* + Unsoaked + N6 medium
11.	T_11_	*G2* + Soaked + MS medium
12.	T_12_	*G2* + Soaked + ½ MS medium
13.	T_13_	*G2* + Soaked + MS + AC medium
14.	T_14_	*G2* + Soaked + Y3 medium
15.	T_15_	*G2* + Soaked + N6 medium
16.	T_16_	*G2* + Unsoaked + MS medium
17.	T_17_	*G2* + Unsoaked + ½ MS medium
18.	T_18_	*G2* + Unsoaked + MS + AC medium
19.	T_19_	*G2* + Unsoaked + Y3 medium
20.	T_20_	*G2* + Unsoaked + N6 medium

### 2.3 Observations recorded

#### 2.3.1 Mean time taken for germination of oil palm ZEs

Mean germination time (MGT) was calculated according to the equation given by Moradi et al., 2008 [8].

MGT=∑Dn/∑n

Where n is the number of zygotic embryos, germinated on day D, and D is the number of days counted from the beginning of germination.

#### 2.3.2 Speed of germination index of oil palm ZEs

This was calculated as described in the Association of Official Seed Analyst (1983) [9] as follow:

$$\text{SGI}=\frac{\text{Number of germinated zygotic embryos}}{\text{Days of first count}}\begin{array}{c} +\text{ ... }+\\ +\;---\;+ \end{array}\frac{\text{Number of germinated zygotic embryos}}{\text{Days of final count}}$$

#### 2.3.3 Speed of shoot formation index of oil palm ZEs

The speed of shoot formation index (SSI) was obtained from

**Figure:**
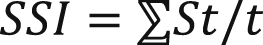

where St was number of shoots formed per culture tube on the day t [10].

#### 2.3.4 Speed of root formation index of oil palm ZEs

The speed of root formation index (SRI) was obtained from

**Figure:**
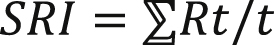

Where Rt was number of roots formed per culture tube on day t [10].

#### 2.3.5 Speed of shoot and root formation index (SSRI)

The speed of shoot and root formation index (SSRI) was obtained from

**Figure:**
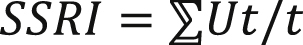

Where Ut was number of shoots and roots formed per culture tube on day t [10].

### 2.4 Data analysis

Data were subjected to analysis of variance (ANOVA) and means were compared by F test, at 5% probability, using ICAR-WASP 2.0 programme developed by ICAR-Central Coastal Agricultural Research Institute, Goa, India (http://www.ccari.res.in/wasp2.0/index.php).

The analysis of variance for each character was carried out as indicated below:

**Table:** 

Anova Table				
Source of variation	df	SS	MSS	F ratio
Replications	(r−1) = (3−1) = 2	RSS	RMSS	RMSS/EMSS
Factor A	(a−1) = (2−1) = 1	ASS	AMSS	AMSS/EMSS
Factor B	(b−1) = (2−1) = 1	BSS	BMSS	BMSS/EMSS
Factor C	(c−1) = (5−1) = 4	CSS	CMSS	CMSS/EMSS
A x B	(a−1) (b−1) = (2−1) (2−1) = 1	ABSS	ABMSS	ABMSS/EMSS
A x C	(a−1) (c−1) = (2−1) (5−1) = 4	ACSS	ACMSS	ACMSS/EMSS
B x C	(b−1) (c−1) = (5−1) (2−1) = 4	BCSS	BCMSS	BCMSS/EMSS
A x B x C	(a−1) (b−1) (c−1) = (2−1) (2−1) (5−1) = 4	ABCSS	ABCMSS	ABCMSS/EMSS
Error	(abc−1) (t−1) = [(2 × 2 × 5)−1] = 19	ESS	EMSS	–
Total	(abcr−1) = [(2 × 2 × 5 × 3)−1]	TSS	–	–

Where,

r = Number of replications.

a = Number of genotypes.

b = Number of pretreatments.

c = Number of culture media.

df = degrees of freedom.

SS = sum of squares.

MSS = Mean sum of squares.

RSS = Replication sum of squares.

ASS = Treatment sum of squares of genotypes.

BSS = Treatment sum of squares of pretreatments.

CSS = Treatment sum of squares of culture media.

ABSS = Treatment sum of squares of genotype × pretreatment interaction.

BCSS = Treatment sum of squares of pretreatment × culture medium interaction.

ACSS = Treatment sum of squares of genotype × culture medium interaction.

ABCSS = Treatment sum of squares of genotype × pretreatment × culture medium interaction.

ESS = Error sum of squares.

TSS = Total sum of squares.

RMSS = Mean sum of squares due to replications.

AMSS = Mean sum of squares due to genotypes.

BMSS = Mean sum of squares due to pretreatments.

CMSS = Mean sum of squares due to culture media.

ABMSS = Mean sum of squares due to genotype × pretreatment interaction.

ACMSS = Mean sum of squares due to pretreatment × culture medium interaction.

BCMSS = Mean sum of squares due to genotype × culture medium interaction.

ABCMSS = Mean sum of squares due to genotype × pretreatment × culture medium interaction.

EMSS = Mean sum of squares due to error.

The test of significance was carried out by ‘F’ table values given by Fisher and Yates (1963).
