# Supplementary material for: Data on germination, growth and morphological changes of oil palm (Elaeis guineensis Jacq.) zygotic embryos during in vitro culturing
Source: Data Brief. 2019 Dec 16;28:104975. doi: 10.1016/j.dib.2019.104975 (PMC7093796; doi:10.1016/j.dib.2019.104975)
Supplement: Multimedia component 2 [file mmc2.zip › dib_104975_G1 Block_sscnars8 GER GL_V2_mmc2.RTF]

CENTER: NA
Experiment No: NACrop: NA
Year: NASeason: NA
Block Design Experiment at NA For NA
ANOVA Analysis - Dependent Variable is MGT

Source	DF	Type III SS	Mean Square	F Value	Pr > F	Significant	
Rep	2	15.4196	7.7098	1.5497	0.2393	NS	
Trt	9	52.7722	5.8636	1.1786	0.3645	NS	
Error	18	89.5505	4.9750	.	.	-	
Corrected Total	29	157.7423	.	.	.	-	
** - Significant at 1%, * - Significant  at 5%, NS - Non Significant	


Analysis Performed at IASRI Server


Block Design Experiment at NA For NA
ANOVA Analysis - Dependent Variable is SGI

Source	DF	Type III SS	Mean Square	F Value	Pr > F	Significant	
Rep	2	2.6678	1.3339	2.1517	0.1453	NS	
Trt	9	30.1745	3.3527	5.4081	0.0012	**	
Error	18	11.1590	0.6199	.	.	-	
Corrected Total	29	44.0013	.	.	.	-	
** - Significant at 1%, * - Significant  at 5%, NS - Non Significant	


Analysis Performed at IASRI Server


Block Design Experiment at NA For NA
ANOVA Analysis - Dependent Variable is SVI_I

Source	DF	Type III SS	Mean Square	F Value	Pr > F	Significant	
Rep	2	0.2101	0.1051	0.2581	0.7753	NS	
Trt	9	17.3190	1.9243	4.7279	0.0025	**	
Error	18	7.3262	0.4070	.	.	-	
Corrected Total	29	24.8554	.	.	.	-	
** - Significant at 1%, * - Significant  at 5%, NS - Non Significant	


Analysis Performed at IASRI Server


Block Design Experiment at NA For NA
ANOVA Analysis - Dependent Variable is SVI__ii

Source	DF	Type III SS	Mean Square	F Value	Pr > F	Significant	
Rep	2	2.1919	1.0960	0.1614	0.8522	NS	
Trt	9	250.6229	27.8470	4.1002	0.0053	**	
Error	18	122.2479	6.7916	.	.	-	
Corrected Total	29	375.0627	.	.	.	-	
** - Significant at 1%, * - Significant  at 5%, NS - Non Significant	


Analysis Performed at IASRI Server


Block Design Experiment at NA For NA
ANOVA Analysis - Dependent Variable is _10s5

Source	DF	Type III SS	Mean Square	F Value	Pr > F	Significant	
Rep	2	102.2404	51.1202	1.2236	0.3175	NS	
Trt	9	3120.5810	346.7312	8.2993	0.0001	**	
Error	18	752.0147	41.7786	.	.	-	
Corrected Total	29	3974.8361	.	.	.	-	
** - Significant at 1%, * - Significant  at 5%, NS - Non Significant	


Analysis Performed at IASRI Server


Block Design Experiment at NA For NA
ANOVA Analysis - Dependent Variable is _1s0

Source	DF	Type III SS	Mean Square	F Value	Pr > F	Significant	
Rep	2	19.0112	9.5056	0.5124	0.6076	NS	
Trt	9	1108.6072	123.1786	6.6395	0.0003	**	
Error	18	333.9430	18.5524	.	.	-	
Corrected Total	29	1461.5614	.	.	.	-	
** - Significant at 1%, * - Significant  at 5%, NS - Non Significant	


Analysis Performed at IASRI Server


Block Design Experiment at NA For NA
ANOVA Analysis - Dependent Variable is _1s1

Source	DF	Type III SS	Mean Square	F Value	Pr > F	Significant	
Rep	2	102.0452	51.0226	9.5834	0.0015	**	
Trt	9	800.9110	88.9901	16.7147	<.0001	**	
Error	18	95.8331	5.3241	.	.	-	
Corrected Total	29	998.7893	.	.	.	-	
** - Significant at 1%, * - Significant  at 5%, NS - Non Significant	


Analysis Performed at IASRI Server


Block Design Experiment at NA For NA
ANOVA Analysis - Dependent Variable is _1s2

Source	DF	Type III SS	Mean Square	F Value	Pr > F	Significant	
Rep	2	181.5614	90.7807	4.7822	0.0216	*	
Trt	9	268.7694	29.8633	1.5732	0.1974	NS	
Error	18	341.6936	18.9830	.	.	-	
Corrected Total	29	792.0245	.	.	.	-	
** - Significant at 1%, * - Significant  at 5%, NS - Non Significant	


Analysis Performed at IASRI Server


Block Design Experiment at NA For NA
ANOVA Analysis - Dependent Variable is _2s0

Source	DF	Type III SS	Mean Square	F Value	Pr > F	Significant	
Rep	2	10.1281	5.0640	0.4511	0.6439	NS	
Trt	9	1732.6325	192.5147	17.1508	<.0001	**	
Error	18	202.0463	11.2248	.	.	-	
Corrected Total	29	1944.8069	.	.	.	-	
** - Significant at 1%, * - Significant  at 5%, NS - Non Significant	


Analysis Performed at IASRI Server


Block Design Experiment at NA For NA
ANOVA Analysis - Dependent Variable is _2s1

Source	DF	Type III SS	Mean Square	F Value	Pr > F	Significant	
Rep	2	35.1755	17.5878	0.4225	0.6617	NS	
Trt	9	1213.4544	134.8283	3.2389	0.0162	*	
Error	18	749.3063	41.6281	.	.	-	
Corrected Total	29	1997.9362	.	.	.	-	
** - Significant at 1%, * - Significant  at 5%, NS - Non Significant	


Analysis Performed at IASRI Server


Block Design Experiment at NA For NA
ANOVA Analysis - Dependent Variable is _2s2

Source	DF	Type III SS	Mean Square	F Value	Pr > F	Significant	
Rep	2	55.4165	27.7083	0.7575	0.4832	NS	
Trt	9	1024.2519	113.8058	3.1114	0.0193	*	
Error	18	658.3952	36.5775	.	.	-	
Corrected Total	29	1738.0636	.	.	.	-	
** - Significant at 1%, * - Significant  at 5%, NS - Non Significant	


Analysis Performed at IASRI Server


Block Design Experiment at NA For NA
ANOVA Analysis - Dependent Variable is _2s3

Source	DF	Type III SS	Mean Square	F Value	Pr > F	Significant	
Rep	2	57.0984	28.5492	0.7442	0.4892	NS	
Trt	9	3351.1245	372.3472	9.7063	<.0001	**	
Error	18	690.5026	38.3613	.	.	-	
Corrected Total	29	4098.7255	.	.	.	-	
** - Significant at 1%, * - Significant  at 5%, NS - Non Significant	


Analysis Performed at IASRI Server


Block Design Experiment at NA For NA
ANOVA Analysis - Dependent Variable is _3s0

Source	DF	Type III SS	Mean Square	F Value	Pr > F	Significant	
Rep	2	45.0245	22.5123	0.8102	0.4604	NS	
Trt	9	1858.1914	206.4657	7.4301	0.0002	**	
Error	18	500.1781	27.7877	.	.	-	
Corrected Total	29	2403.3939	.	.	.	-	
** - Significant at 1%, * - Significant  at 5%, NS - Non Significant	


Analysis Performed at IASRI Server


Block Design Experiment at NA For NA
ANOVA Analysis - Dependent Variable is _3s1

Source	DF	Type III SS	Mean Square	F Value	Pr > F	Significant	
Rep	2	34.7132	17.3566	0.8526	0.4428	NS	
Trt	9	343.0657	38.1184	1.8724	0.1231	NS	
Error	18	366.4512	20.3584	.	.	-	
Corrected Total	29	744.2300	.	.	.	-	
** - Significant at 1%, * - Significant  at 5%, NS - Non Significant	


Analysis Performed at IASRI Server


Block Design Experiment at NA For NA
ANOVA Analysis - Dependent Variable is _3s2

Source	DF	Type III SS	Mean Square	F Value	Pr > F	Significant	
Rep	2	962.5213	481.2607	7.3461	0.0046	**	
Trt	9	1008.1838	112.0204	1.7099	0.1590	NS	
Error	18	1179.2186	65.5121	.	.	-	
Corrected Total	29	3149.9237	.	.	.	-	
** - Significant at 1%, * - Significant  at 5%, NS - Non Significant	


Analysis Performed at IASRI Server


Block Design Experiment at NA For NA
ANOVA Analysis - Dependent Variable is _3s3

Source	DF	Type III SS	Mean Square	F Value	Pr > F	Significant	
Rep	2	17.6488	8.8244	0.4656	0.6351	NS	
Trt	9	1185.9997	131.7777	6.9524	0.0003	**	
Error	18	341.1749	18.9542	.	.	-	
Corrected Total	29	1544.8234	.	.	.	-	
** - Significant at 1%, * - Significant  at 5%, NS - Non Significant	


Analysis Performed at IASRI Server


Block Design Experiment at NA For NA
ANOVA Analysis - Dependent Variable is _3s4

Source	DF	Type III SS	Mean Square	F Value	Pr > F	Significant	
Rep	2	54.5693	27.2847	0.5738	0.5733	NS	
Trt	9	1080.4536	120.0504	2.5247	0.0451	*	
Error	18	855.9008	47.5500	.	.	-	
Corrected Total	29	1990.9237	.	.	.	-	
** - Significant at 1%, * - Significant  at 5%, NS - Non Significant	


Analysis Performed at IASRI Server


Block Design Experiment at NA For NA
ANOVA Analysis - Dependent Variable is _4s0

Source	DF	Type III SS	Mean Square	F Value	Pr > F	Significant	
Rep	2	20.1410	10.0705	0.3743	0.6930	NS	
Trt	9	1622.5763	180.2863	6.7003	0.0003	**	
Error	18	484.3284	26.9071	.	.	-	
Corrected Total	29	2127.0458	.	.	.	-	
** - Significant at 1%, * - Significant  at 5%, NS - Non Significant	


Analysis Performed at IASRI Server


Block Design Experiment at NA For NA
ANOVA Analysis - Dependent Variable is _4s1

Source	DF	Type III SS	Mean Square	F Value	Pr > F	Significant	
Rep	2	26.1625	13.0813	0.2694	0.7668	NS	
Trt	9	675.5149	75.0572	1.5459	0.2061	NS	
Error	18	873.9506	48.5528	.	.	-	
Corrected Total	29	1575.6280	.	.	.	-	
** - Significant at 1%, * - Significant  at 5%, NS - Non Significant	


Analysis Performed at IASRI Server


Block Design Experiment at NA For NA
ANOVA Analysis - Dependent Variable is _4s2

Source	DF	Type III SS	Mean Square	F Value	Pr > F	Significant	
Rep	2	65.0831	32.5415	0.4550	0.6416	NS	
Trt	9	1851.7540	205.7504	2.8766	0.0270	*	
Error	18	1287.4586	71.5255	.	.	-	
Corrected Total	29	3204.2957	.	.	.	-	
** - Significant at 1%, * - Significant  at 5%, NS - Non Significant	


Analysis Performed at IASRI Server


Block Design Experiment at NA For NA
ANOVA Analysis - Dependent Variable is _4s3

Source	DF	Type III SS	Mean Square	F Value	Pr > F	Significant	
Rep	2	53.3792	26.6896	1.2139	0.3202	NS	
Trt	9	563.8862	62.6540	2.8496	0.0280	*	
Error	18	395.7674	21.9871	.	.	-	
Corrected Total	29	1013.0328	.	.	.	-	
** - Significant at 1%, * - Significant  at 5%, NS - Non Significant	


Analysis Performed at IASRI Server


Block Design Experiment at NA For NA
ANOVA Analysis - Dependent Variable is _4s4

Source	DF	Type III SS	Mean Square	F Value	Pr > F	Significant	
Rep	2	41.5610	20.7805	0.7499	0.4866	NS	
Trt	9	1360.0478	151.1164	5.4536	0.0011	**	
Error	18	498.7750	27.7097	.	.	-	
Corrected Total	29	1900.3837	.	.	.	-	
** - Significant at 1%, * - Significant  at 5%, NS - Non Significant	


Analysis Performed at IASRI Server


Block Design Experiment at NA For NA
ANOVA Analysis - Dependent Variable is _4s5

Source	DF	Type III SS	Mean Square	F Value	Pr > F	Significant	
Rep	2	22.6565	11.3282	0.4737	0.6302	NS	
Trt	9	181.2519	20.1391	0.8421	0.5887	NS	
Error	18	430.4733	23.9152	.	.	-	
Corrected Total	29	634.3817	.	.	.	-	
** - Significant at 1%, * - Significant  at 5%, NS - Non Significant	


Analysis Performed at IASRI Server


Block Design Experiment at NA For NA
ANOVA Analysis - Dependent Variable is _5s0

Source	DF	Type III SS	Mean Square	F Value	Pr > F	Significant	
Rep	2	12.9950	6.4975	0.2342	0.7936	NS	
Trt	9	1626.3653	180.7073	6.5141	0.0004	**	
Error	18	499.3404	27.7411	.	.	-	
Corrected Total	29	2138.7007	.	.	.	-	
** - Significant at 1%, * - Significant  at 5%, NS - Non Significant	


Analysis Performed at IASRI Server


Block Design Experiment at NA For NA
ANOVA Analysis - Dependent Variable is _5s1

Source	DF	Type III SS	Mean Square	F Value	Pr > F	Significant	
Rep	2	19.8893	9.9447	0.1997	0.8207	NS	
Trt	9	717.7844	79.7538	1.6018	0.1886	NS	
Error	18	896.2200	49.7900	.	.	-	
Corrected Total	29	1633.8937	.	.	.	-	
** - Significant at 1%, * - Significant  at 5%, NS - Non Significant	


Analysis Performed at IASRI Server


Block Design Experiment at NA For NA
ANOVA Analysis - Dependent Variable is _5s2

Source	DF	Type III SS	Mean Square	F Value	Pr > F	Significant	
Rep	2	330.1544	165.0772	4.5556	0.0251	*	
Trt	9	647.0684	71.8965	1.9841	0.1033	NS	
Error	18	652.2520	36.2362	.	.	-	
Corrected Total	29	1629.4747	.	.	.	-	
** - Significant at 1%, * - Significant  at 5%, NS - Non Significant	


Analysis Performed at IASRI Server


Block Design Experiment at NA For NA
ANOVA Analysis - Dependent Variable is _5s3

Source	DF	Type III SS	Mean Square	F Value	Pr > F	Significant	
Rep	2	186.7693	93.3846	4.0867	0.0344	*	
Trt	9	1074.7464	119.4163	5.2259	0.0014	**	
Error	18	411.3143	22.8508	.	.	-	
Corrected Total	29	1672.8300	.	.	.	-	
** - Significant at 1%, * - Significant  at 5%, NS - Non Significant	


Analysis Performed at IASRI Server


Block Design Experiment at NA For NA
ANOVA Analysis - Dependent Variable is _5s4

Source	DF	Type III SS	Mean Square	F Value	Pr > F	Significant	
Rep	2	89.3888	44.6944	2.3614	0.1228	NS	
Trt	9	1392.8741	154.7638	8.1768	0.0001	**	
Error	18	340.6913	18.9273	.	.	-	
Corrected Total	29	1822.9542	.	.	.	-	
** - Significant at 1%, * - Significant  at 5%, NS - Non Significant	


Analysis Performed at IASRI Server


Block Design Experiment at NA For NA
ANOVA Analysis - Dependent Variable is _5s5

Source	DF	Type III SS	Mean Square	F Value	Pr > F	Significant	
Rep	2	84.4616	42.2308	1.3521	0.2837	NS	
Trt	9	820.3207	91.1467	2.9183	0.0254	*	
Error	18	562.1931	31.2330	.	.	-	
Corrected Total	29	1466.9754	.	.	.	-	
** - Significant at 1%, * - Significant  at 5%, NS - Non Significant	


Analysis Performed at IASRI Server


Block Design Experiment at NA For NA
ANOVA Analysis - Dependent Variable is _6s5

Source	DF	Type III SS	Mean Square	F Value	Pr > F	Significant	
Rep	2	9.3309	4.6654	0.1284	0.8803	NS	
Trt	9	1403.2807	155.9201	4.2901	0.0042	**	
Error	18	654.2014	36.3445	.	.	-	
Corrected Total	29	2066.8129	.	.	.	-	
** - Significant at 1%, * - Significant  at 5%, NS - Non Significant	


Analysis Performed at IASRI Server


Block Design Experiment at NA For NA
ANOVA Analysis - Dependent Variable is _7s5

Source	DF	Type III SS	Mean Square	F Value	Pr > F	Significant	
Rep	2	112.1801	56.0900	1.2513	0.3099	NS	
Trt	9	1463.5541	162.6171	3.6279	0.0096	**	
Error	18	806.8304	44.8239	.	.	-	
Corrected Total	29	2382.5646	.	.	.	-	
** - Significant at 1%, * - Significant  at 5%, NS - Non Significant	


Analysis Performed at IASRI Server


Block Design Experiment at NA For NA
ANOVA Analysis - Dependent Variable is _8s5

Source	DF	Type III SS	Mean Square	F Value	Pr > F	Significant	
Rep	2	61.4811	30.7405	0.6236	0.5472	NS	
Trt	9	1594.6631	177.1848	3.5942	0.0100	**	
Error	18	887.3654	49.2981	.	.	-	
Corrected Total	29	2543.5095	.	.	.	-	
** - Significant at 1%, * - Significant  at 5%, NS - Non Significant	


Analysis Performed at IASRI Server


Block Design Experiment at NA For NA
ANOVA Analysis - Dependent Variable is _9s5

Source	DF	Type III SS	Mean Square	F Value	Pr > F	Significant	
Rep	2	121.9632	60.9816	1.1795	0.3301	NS	
Trt	9	2499.1149	277.6794	5.3707	0.0012	**	
Error	18	930.6413	51.7023	.	.	-	
Corrected Total	29	3551.7193	.	.	.	-	
** - Significant at 1%, * - Significant  at 5%, NS - Non Significant	


Analysis Performed at IASRI Server


Block Design Experiment at NA For NA
Treatment Mean Table

Trt	Mgt	Sgi	Svi_i	
Treatment Name	Treatment Description	Treatment of Mgt	Rank of Treatment	Treatment of Sgi	Rank of Treatment	Treatment of Svi_i	Rank of Treatment	
1		16.17	2	4.87CDE               	8	2.71BC                	7	
2		14.85	5	5.80BCD               	6	3.47AB                	5	
3		16.85	1	4.01E                 	10	2.14C                 	10	
4		13.29	9	6.92AB                	2	3.80AB                	3	
5		12.25	10	7.30A                 	1	4.08A                 	2	
6		14.42	6	4.90CDE               	7	2.26C                 	8	
7		13.51	8	4.46DE                	9	2.23C                 	9	
8		14.18	7	5.97ABC               	5	4.34A                 	1	
9		14.90	4	6.07ABC               	3	3.48AB                	4	
10		15.74	3	6.01ABC               	4	3.36AB                	6	
General Mean		14.62	.	5.63	.	3.19	.	
p-Value		0.3645	.	0.0012	.	0.0025	.	
CV(%)		15.26	.	13.98	.	20.02	.	
SE(d)		1.821	.	0.643	.	0.521	.	
LSD at 5%		NS	.	1.3506	.	1.0944	.	

Svi__ii	_10s5	_1s0	_1s1	_1s2	
Treatment of Svi__ii	Rank of Treatment	Treatment of _10s5	Rank of Treatment	Treatment of _1s0	Rank of Treatment	Treatment of _1s1	Rank of Treatment	Treatment of _1s2	Rank of Treatment	
7.12CDE               	8	0.00C                 	9	57.91A                 	2	25.31C                 	9	18.05	10	
8.80BCDE              	6	14.76B                 	7	42.13C                 	8	34.15AB                	6	28.67	2	
8.29CDE               	7	31.78A                 	2	58.07A                 	1	21.34D                 	10	21.90	9	
11.37ABC               	4	18.05B                 	3	45.03C                 	5	34.18AB                	3	25.00	4	
13.16AB                	2	15.00B                 	6	42.12C                 	9	34.18AB                	4	28.86	1	
6.33DE                	9	-0.00C                 	10	47.88BC                	4	33.16B                 	7	22.60	8	
5.86E                 	10	12.29B                 	8	53.76AB                	3	25.31C                 	8	23.86	7	
15.12A                 	1	32.09A                 	1	45.00C                 	6	34.18AB                	5	25.00	5	
11.53ABC               	3	16.60B                 	5	44.03C                 	7	36.24AB                	2	24.05	6	
10.55BCD               	5	16.60B                 	4	42.12C                 	10	37.26A                 	1	25.19	3	
9.81	.	15.72	.	47.81	.	31.53	.	24.32	.	
0.0053	.	<.0001	.	0.0003	.	<.0001	.	0.1974	.	
26.56	.	41.13	.	9.01	.	7.32	.	17.92	.	
2.128	.	5.278	.	3.517	.	1.884	.	3.557	.	
4.4704	.	11.088	.	7.3886	.	3.9581	.	NS	.	

_2s0	_2s1	_2s2	_2s3	
Treatment of _2s0	Rank of Treatment	Treatment of _2s1	Rank of Treatment	Treatment of _2s2	Rank of Treatment	Treatment of _2s3	Rank of Treatment	
36.24B                 	3	25.00BCDE              	6	36.24A                 	1	19.50CD                	9	
33.16BC                	5	34.15AB                	2	35.25AB                	2	10.45D                 	10	
48.93A                 	1	18.61DE                	9	17.60D                 	10	24.31C                 	8	
25.31D                 	9	23.53BCDE              	7	28.54ABC               	4	45.96A                 	1	
24.05D                 	10	14.76E                 	10	30.95ABC               	3	44.99A                 	2	
36.24B                 	4	28.86ABCD              	4	24.05CD                	7	29.93BC                	5	
44.04A                 	2	21.34CDE               	8	21.34CD                	8	29.93BC                	6	
27.71CD                	8	29.93ABC               	3	21.34CD                	9	39.21AB                	3	
32.14BC                	6	25.31ABCDE             	5	25.31BCD               	5	36.24AB                	4	
28.86CD                	7	36.24A                 	1	24.05CD                	6	29.93BC                	7	
33.67	.	25.77	.	26.46	.	31.04	.	
<.0001	.	0.0162	.	0.0193	.	<.0001	.	
9.95	.	25.04	.	22.85	.	19.95	.	
2.736	.	5.268	.	4.938	.	5.057	.	
5.7472	.	11.068	.	10.375	.	10.625	.	

_3s0	_3s1	_3s2	_3s3	_3s4	
Treatment of _3s0	Rank of Treatment	Treatment of _3s1	Rank of Treatment	Treatment of _3s2	Rank of Treatment	Treatment of _3s3	Rank of Treatment	Treatment of _3s4	Rank of Treatment	
31.07CD                	4	23.86	2	12.92	5	39.21AB                	3	19.50C                 	10	
24.31D                 	7	24.05	1	18.05	4	28.86CDE               	7	34.81AB                	2	
46.05A                 	1	17.47	5	10.45	7	23.16E                 	10	23.16BC                	9	
22.60D                 	9	16.60	7	20.26	3	30.95CD                	6	32.30AB                	3	
22.79D                 	8	12.92	10	8.61	10	34.18BC                	5	42.12A                 	1	
34.18BC                	3	18.05	3	26.45	1	25.31DE                	9	26.45BC                	7	
41.15AB                	2	16.21	8	10.45	8	28.78CDE               	8	26.45BC                	8	
26.45CD                	5	18.05	4	10.45	9	41.15AB                	2	27.60BC                	6	
26.45CD                	6	14.76	9	21.34	2	36.24ABC               	4	29.93BC                	5	
22.60D                 	10	16.60	6	10.45	6	42.12A                 	1	31.07ABC               	4	
29.76	.	17.85	.	14.94	.	33.00	.	29.34	.	
0.0002	.	0.1231	.	0.1590	.	0.0003	.	0.0451	.	
17.71	.	25.27	.	54.16	.	13.19	.	23.50	.	
4.304	.	3.684	.	6.609	.	3.555	.	5.630	.	
9.0425	.	NS	.	NS	.	7.4682	.	11.829	.	

_4s0	_4s1	_4s2	_4s3	_4s4	
Treatment of _4s0	Rank of Treatment	Treatment of _4s1	Rank of Treatment	Treatment of _4s2	Rank of Treatment	Treatment of _4s3	Rank of Treatment	Treatment of _4s4	Rank of Treatment	
30.00BC                	4	18.05	2	24.98A                 	1	30.95A                 	1	33.16D                 	9	
23.16C                 	7	22.79	1	12.92ABC               	3	25.31ABC               	6	41.12BCD               	6	
43.08A                 	1	12.92	7	4.31C                 	7	21.34CD                	8	35.68CD                	8	
22.60C                 	10	6.14	9	0.00C                 	10	28.67ABC               	3	49.80AB                	2	
22.79C                 	8	12.92	8	4.31C                 	8	27.22ABC               	4	48.87AB                	3	
34.18AB                	3	16.21	3	22.60AB                	2	22.60BCD               	7	33.04D                 	10	
41.15A                 	2	14.76	5	4.31C                 	9	20.76CD                	9	36.24CD                	7	
26.45BC                	6	13.74	6	6.14C                 	6	16.60D                 	10	50.79A                 	1	
26.45BC                	5	6.14	10	10.45BC                	4	29.93AB                	2	43.08ABC               	5	
22.60C                 	9	14.76	4	6.14C                 	5	26.45ABC               	5	47.88AB                	4	
29.25	.	13.84	.	9.62	.	24.98	.	41.97	.	
0.0003	.	0.2061	.	0.0270	.	0.0280	.	0.0011	.	
17.74	.	50.33	.	87.95	.	18.77	.	12.54	.	
4.235	.	5.689	.	6.906	.	3.829	.	4.298	.	
8.8981	.	NS	.	14.508	.	8.0436	.	9.0298	.	

_4s5	_5s0	_5s1	_5s2	_5s3	
Treatment of _4s5	Rank of Treatment	Treatment of _5s0	Rank of Treatment	Treatment of _5s1	Rank of Treatment	Treatment of _5s2	Rank of Treatment	Treatment of _5s3	Rank of Treatment	
0.00	5	27.71BC                	4	19.50	2	10.45	2	27.71A                 	1	
0.00	6	23.16C                 	7	22.79	1	4.31	5	22.79AB                	4	
6.14	1	43.08A                 	1	12.92	7	4.31	6	10.45CD                	9	
0.00	7	22.60C                 	9	6.14	9	0.00	9	26.26A                 	2	
0.00	8	22.79C                 	8	12.92	8	4.31	8	21.14AB                	7	
0.00	9	34.18AB                	3	16.21	3	16.60	1	22.60AB                	6	
0.00	10	41.15A                 	2	14.76	5	6.14	3	18.05BC                	8	
6.14	2	26.45BC                	5	13.74	6	4.31	7	8.61D                 	10	
0.00	4	26.45BC                	6	6.14	10	0.00	10	23.74AB                	3	
0.00	3	22.60C                 	10	14.76	4	4.31	4	22.60AB                	5	
1.23	.	29.02	.	13.99	.	5.47	.	20.39	.	
0.5887	.	0.0004	.	0.1886	.	0.1033	.	0.0014	.	
397.91	.	18.15	.	50.44	.	109.99	.	23.44	.	
3.993	.	4.300	.	5.761	.	4.915	.	3.903	.	
NS	.	9.035	.	NS	.	NS	.	8.2	.	

_5s4	_5s5	_6s5	_7s5	_8s5	
Treatment of _5s4	Rank of Treatment	Treatment of _5s5	Rank of Treatment	Treatment of _6s5	Rank of Treatment	Treatment of _7s5	Rank of Treatment	Treatment of _8s5	Rank of Treatment	
39.21CD                	8	0.00B                 	9	0.00C                 	7	-0.00B                 	10	0.00B                 	9	
46.00BC                	6	0.00B                 	6	4.31C                 	4	4.31B                 	6	4.31B                 	8	
40.11CD                	7	8.86AB                	2	19.31A                 	1	20.76A                 	1	22.02A                 	2	
51.81AB                	3	4.31B                 	3	4.31C                 	5	8.61B                 	3	10.45AB                	3	
53.76A                 	2	0.00B                 	7	0.00C                 	8	4.31B                 	7	8.61B                 	5	
37.12D                 	10	0.00B                 	10	0.00C                 	9	0.00B                 	9	0.00B                 	10	
37.26D                 	9	0.00B                 	8	8.61BC                	3	8.61B                 	4	8.61B                 	6	
54.89A                 	1	16.21A                 	1	17.47AB                	2	20.45A                 	2	22.29A                 	1	
50.79AB                	5	0.00B                 	4	0.00C                 	10	4.31B                 	8	8.61B                 	4	
51.76AB                	4	0.00B                 	5	4.31C                 	6	8.61B                 	5	8.61B                 	7	
46.27	.	2.94	.	5.83	.	8.00	.	9.35	.	
<.0001	.	0.0254	.	0.0042	.	0.0096	.	0.0100	.	
9.40	.	190.27	.	103.39	.	83.72	.	75.08	.	
3.552	.	4.563	.	4.923	.	5.467	.	5.733	.	
7.4629	.	9.5867	.	10.342	.	11.485	.	12.044	.	

_9s5	
Treatment of _9s5	Rank of Treatment	
0.00C                 	9	
12.92B                 	6	
29.31A                 	1	
18.05AB                	3	
13.74B                 	5	
-0.00C                 	10	
12.29BC                	7	
27.52A                 	2	
12.29BC                	8	
14.76B                 	4	
14.09	.	
0.0012	.	
51.04	.	
5.871	.	
12.334	.	


Means with atleast one letter common are not statistically significant using
Fisher's Least Significant Difference

Grouping letters on treatments were made using pdglm800.sas which can be downloaded from http://animalscience.ag.utk.edu/FacultyStaff/ArnoldSaxton.html#software
Analysis Performed at IASRI Server


Block Design Experiment at NA For NA
Treatment Details Table

Obs	Treatment Name	Treatment Details	
1	1		
2	2		
3	3		
4	4		
5	5		
6	6		
7	7		
8	8		
9	9		
10	10		


Analysis Performed at IASRI Server
