# Supplementary material for: Data on germination, growth and morphological changes of oil palm (Elaeis guineensis Jacq.) zygotic embryos during in vitro culturing
Source: Data Brief. 2019 Dec 16;28:104975. doi: 10.1016/j.dib.2019.104975 (PMC7093796; doi:10.1016/j.dib.2019.104975)
Supplement: Multimedia component 3 [file mmc3.zip › dib_104975_G1 Block_sscnars8 GER_V2_mmc3.RTF]

CENTER: NA
Experiment No: NACrop: NA
Year: NASeason: NA
Block Design Experiment at NA For NA
ANOVA Analysis - Dependent Variable is MGT

Source	DF	Type III SS	Mean Square	F Value	Pr > F	Significant	
Rep	2	15.4196	7.7098	1.5497	0.2393	NS	
Trt	9	52.7722	5.8636	1.1786	0.3645	NS	
Error	18	89.5505	4.9750	.	.	-	
Corrected Total	29	157.7423	.	.	.	-	
** - Significant at 1%, * - Significant  at 5%, NS - Non Significant	


Analysis Performed at IASRI Server


Block Design Experiment at NA For NA
ANOVA Analysis - Dependent Variable is SGI

Source	DF	Type III SS	Mean Square	F Value	Pr > F	Significant	
Rep	2	2.6678	1.3339	2.1517	0.1453	NS	
Trt	9	30.1745	3.3527	5.4081	0.0012	**	
Error	18	11.1590	0.6199	.	.	-	
Corrected Total	29	44.0013	.	.	.	-	
** - Significant at 1%, * - Significant  at 5%, NS - Non Significant	


Analysis Performed at IASRI Server


Block Design Experiment at NA For NA
ANOVA Analysis - Dependent Variable is SVI_I

Source	DF	Type III SS	Mean Square	F Value	Pr > F	Significant	
Rep	2	0.2101	0.1051	0.2581	0.7753	NS	
Trt	9	17.3190	1.9243	4.7279	0.0025	**	
Error	18	7.3262	0.4070	.	.	-	
Corrected Total	29	24.8554	.	.	.	-	
** - Significant at 1%, * - Significant  at 5%, NS - Non Significant	


Analysis Performed at IASRI Server


Block Design Experiment at NA For NA
ANOVA Analysis - Dependent Variable is SVI__ii

Source	DF	Type III SS	Mean Square	F Value	Pr > F	Significant	
Rep	2	2.1919	1.0960	0.1614	0.8522	NS	
Trt	9	250.6229	27.8470	4.1002	0.0053	**	
Error	18	122.2479	6.7916	.	.	-	
Corrected Total	29	375.0627	.	.	.	-	
** - Significant at 1%, * - Significant  at 5%, NS - Non Significant	


Analysis Performed at IASRI Server


Block Design Experiment at NA For NA
ANOVA Analysis - Dependent Variable is _10s5

Source	DF	Type III SS	Mean Square	F Value	Pr > F	Significant	
Rep	2	102.2404	51.1202	1.2236	0.3175	NS	
Trt	9	3120.5810	346.7312	8.2993	0.0001	**	
Error	18	752.0147	41.7786	.	.	-	
Corrected Total	29	3974.8361	.	.	.	-	
** - Significant at 1%, * - Significant  at 5%, NS - Non Significant	


Analysis Performed at IASRI Server


Block Design Experiment at NA For NA
ANOVA Analysis - Dependent Variable is _1s0

Source	DF	Type III SS	Mean Square	F Value	Pr > F	Significant	
Rep	2	19.0112	9.5056	0.5124	0.6076	NS	
Trt	9	1108.6072	123.1786	6.6395	0.0003	**	
Error	18	333.9430	18.5524	.	.	-	
Corrected Total	29	1461.5614	.	.	.	-	
** - Significant at 1%, * - Significant  at 5%, NS - Non Significant	


Analysis Performed at IASRI Server


Block Design Experiment at NA For NA
ANOVA Analysis - Dependent Variable is _1s1

Source	DF	Type III SS	Mean Square	F Value	Pr > F	Significant	
Rep	2	102.0452	51.0226	9.5834	0.0015	**	
Trt	9	800.9110	88.9901	16.7147	<.0001	**	
Error	18	95.8331	5.3241	.	.	-	
Corrected Total	29	998.7893	.	.	.	-	
** - Significant at 1%, * - Significant  at 5%, NS - Non Significant	


Analysis Performed at IASRI Server


Block Design Experiment at NA For NA
ANOVA Analysis - Dependent Variable is _1s2

Source	DF	Type III SS	Mean Square	F Value	Pr > F	Significant	
Rep	2	181.5614	90.7807	4.7822	0.0216	*	
Trt	9	268.7694	29.8633	1.5732	0.1974	NS	
Error	18	341.6936	18.9830	.	.	-	
Corrected Total	29	792.0245	.	.	.	-	
** - Significant at 1%, * - Significant  at 5%, NS - Non Significant	


Analysis Performed at IASRI Server


Block Design Experiment at NA For NA
ANOVA Analysis - Dependent Variable is _2s0

Source	DF	Type III SS	Mean Square	F Value	Pr > F	Significant	
Rep	2	10.1281	5.0640	0.4511	0.6439	NS	
Trt	9	1732.6325	192.5147	17.1508	<.0001	**	
Error	18	202.0463	11.2248	.	.	-	
Corrected Total	29	1944.8069	.	.	.	-	
** - Significant at 1%, * - Significant  at 5%, NS - Non Significant	


Analysis Performed at IASRI Server


Block Design Experiment at NA For NA
ANOVA Analysis - Dependent Variable is _2s1

Source	DF	Type III SS	Mean Square	F Value	Pr > F	Significant	
Rep	2	35.1755	17.5878	0.4225	0.6617	NS	
Trt	9	1213.4544	134.8283	3.2389	0.0162	*	
Error	18	749.3063	41.6281	.	.	-	
Corrected Total	29	1997.9362	.	.	.	-	
** - Significant at 1%, * - Significant  at 5%, NS - Non Significant	


Analysis Performed at IASRI Server


Block Design Experiment at NA For NA
ANOVA Analysis - Dependent Variable is _2s2

Source	DF	Type III SS	Mean Square	F Value	Pr > F	Significant	
Rep	2	55.4165	27.7083	0.7575	0.4832	NS	
Trt	9	1024.2519	113.8058	3.1114	0.0193	*	
Error	18	658.3952	36.5775	.	.	-	
Corrected Total	29	1738.0636	.	.	.	-	
** - Significant at 1%, * - Significant  at 5%, NS - Non Significant	


Analysis Performed at IASRI Server


Block Design Experiment at NA For NA
ANOVA Analysis - Dependent Variable is _2s3

Source	DF	Type III SS	Mean Square	F Value	Pr > F	Significant	
Rep	2	57.0984	28.5492	0.7442	0.4892	NS	
Trt	9	3351.1245	372.3472	9.7063	<.0001	**	
Error	18	690.5026	38.3613	.	.	-	
Corrected Total	29	4098.7255	.	.	.	-	
** - Significant at 1%, * - Significant  at 5%, NS - Non Significant	


Analysis Performed at IASRI Server


Block Design Experiment at NA For NA
ANOVA Analysis - Dependent Variable is _3s0

Source	DF	Type III SS	Mean Square	F Value	Pr > F	Significant	
Rep	2	45.0245	22.5123	0.8102	0.4604	NS	
Trt	9	1858.1914	206.4657	7.4301	0.0002	**	
Error	18	500.1781	27.7877	.	.	-	
Corrected Total	29	2403.3939	.	.	.	-	
** - Significant at 1%, * - Significant  at 5%, NS - Non Significant	


Analysis Performed at IASRI Server


Block Design Experiment at NA For NA
ANOVA Analysis - Dependent Variable is _3s1

Source	DF	Type III SS	Mean Square	F Value	Pr > F	Significant	
Rep	2	34.7132	17.3566	0.8526	0.4428	NS	
Trt	9	343.0657	38.1184	1.8724	0.1231	NS	
Error	18	366.4512	20.3584	.	.	-	
Corrected Total	29	744.2300	.	.	.	-	
** - Significant at 1%, * - Significant  at 5%, NS - Non Significant	


Analysis Performed at IASRI Server


Block Design Experiment at NA For NA
ANOVA Analysis - Dependent Variable is _3s2

Source	DF	Type III SS	Mean Square	F Value	Pr > F	Significant	
Rep	2	962.5213	481.2607	7.3461	0.0046	**	
Trt	9	1008.1838	112.0204	1.7099	0.1590	NS	
Error	18	1179.2186	65.5121	.	.	-	
Corrected Total	29	3149.9237	.	.	.	-	
** - Significant at 1%, * - Significant  at 5%, NS - Non Significant	


Analysis Performed at IASRI Server


Block Design Experiment at NA For NA
ANOVA Analysis - Dependent Variable is _3s3

Source	DF	Type III SS	Mean Square	F Value	Pr > F	Significant	
Rep	2	17.6488	8.8244	0.4656	0.6351	NS	
Trt	9	1185.9997	131.7777	6.9524	0.0003	**	
Error	18	341.1749	18.9542	.	.	-	
Corrected Total	29	1544.8234	.	.	.	-	
** - Significant at 1%, * - Significant  at 5%, NS - Non Significant	


Analysis Performed at IASRI Server


Block Design Experiment at NA For NA
ANOVA Analysis - Dependent Variable is _3s4

Source	DF	Type III SS	Mean Square	F Value	Pr > F	Significant	
Rep	2	54.5693	27.2847	0.5738	0.5733	NS	
Trt	9	1080.4536	120.0504	2.5247	0.0451	*	
Error	18	855.9008	47.5500	.	.	-	
Corrected Total	29	1990.9237	.	.	.	-	
** - Significant at 1%, * - Significant  at 5%, NS - Non Significant	


Analysis Performed at IASRI Server


Block Design Experiment at NA For NA
ANOVA Analysis - Dependent Variable is _4s0

Source	DF	Type III SS	Mean Square	F Value	Pr > F	Significant	
Rep	2	20.1410	10.0705	0.3743	0.6930	NS	
Trt	9	1622.5763	180.2863	6.7003	0.0003	**	
Error	18	484.3284	26.9071	.	.	-	
Corrected Total	29	2127.0458	.	.	.	-	
** - Significant at 1%, * - Significant  at 5%, NS - Non Significant	


Analysis Performed at IASRI Server


Block Design Experiment at NA For NA
ANOVA Analysis - Dependent Variable is _4s1

Source	DF	Type III SS	Mean Square	F Value	Pr > F	Significant	
Rep	2	26.1625	13.0813	0.2694	0.7668	NS	
Trt	9	675.5149	75.0572	1.5459	0.2061	NS	
Error	18	873.9506	48.5528	.	.	-	
Corrected Total	29	1575.6280	.	.	.	-	
** - Significant at 1%, * - Significant  at 5%, NS - Non Significant	


Analysis Performed at IASRI Server


Block Design Experiment at NA For NA
ANOVA Analysis - Dependent Variable is _4s2

Source	DF	Type III SS	Mean Square	F Value	Pr > F	Significant	
Rep	2	65.0831	32.5415	0.4550	0.6416	NS	
Trt	9	1851.7540	205.7504	2.8766	0.0270	*	
Error	18	1287.4586	71.5255	.	.	-	
Corrected Total	29	3204.2957	.	.	.	-	
** - Significant at 1%, * - Significant  at 5%, NS - Non Significant	


Analysis Performed at IASRI Server


Block Design Experiment at NA For NA
ANOVA Analysis - Dependent Variable is _4s3

Source	DF	Type III SS	Mean Square	F Value	Pr > F	Significant	
Rep	2	53.3792	26.6896	1.2139	0.3202	NS	
Trt	9	563.8862	62.6540	2.8496	0.0280	*	
Error	18	395.7674	21.9871	.	.	-	
Corrected Total	29	1013.0328	.	.	.	-	
** - Significant at 1%, * - Significant  at 5%, NS - Non Significant	


Analysis Performed at IASRI Server


Block Design Experiment at NA For NA
ANOVA Analysis - Dependent Variable is _4s4

Source	DF	Type III SS	Mean Square	F Value	Pr > F	Significant	
Rep	2	41.5610	20.7805	0.7499	0.4866	NS	
Trt	9	1360.0478	151.1164	5.4536	0.0011	**	
Error	18	498.7750	27.7097	.	.	-	
Corrected Total	29	1900.3837	.	.	.	-	
** - Significant at 1%, * - Significant  at 5%, NS - Non Significant	


Analysis Performed at IASRI Server


Block Design Experiment at NA For NA
ANOVA Analysis - Dependent Variable is _4s5

Source	DF	Type III SS	Mean Square	F Value	Pr > F	Significant	
Rep	2	22.6565	11.3282	0.4737	0.6302	NS	
Trt	9	181.2519	20.1391	0.8421	0.5887	NS	
Error	18	430.4733	23.9152	.	.	-	
Corrected Total	29	634.3817	.	.	.	-	
** - Significant at 1%, * - Significant  at 5%, NS - Non Significant	


Analysis Performed at IASRI Server


Block Design Experiment at NA For NA
ANOVA Analysis - Dependent Variable is _5s0

Source	DF	Type III SS	Mean Square	F Value	Pr > F	Significant	
Rep	2	12.9950	6.4975	0.2342	0.7936	NS	
Trt	9	1626.3653	180.7073	6.5141	0.0004	**	
Error	18	499.3404	27.7411	.	.	-	
Corrected Total	29	2138.7007	.	.	.	-	
** - Significant at 1%, * - Significant  at 5%, NS - Non Significant	


Analysis Performed at IASRI Server


Block Design Experiment at NA For NA
ANOVA Analysis - Dependent Variable is _5s1

Source	DF	Type III SS	Mean Square	F Value	Pr > F	Significant	
Rep	2	19.8893	9.9447	0.1997	0.8207	NS	
Trt	9	717.7844	79.7538	1.6018	0.1886	NS	
Error	18	896.2200	49.7900	.	.	-	
Corrected Total	29	1633.8937	.	.	.	-	
** - Significant at 1%, * - Significant  at 5%, NS - Non Significant	


Analysis Performed at IASRI Server


Block Design Experiment at NA For NA
ANOVA Analysis - Dependent Variable is _5s2

Source	DF	Type III SS	Mean Square	F Value	Pr > F	Significant	
Rep	2	330.1544	165.0772	4.5556	0.0251	*	
Trt	9	647.0684	71.8965	1.9841	0.1033	NS	
Error	18	652.2520	36.2362	.	.	-	
Corrected Total	29	1629.4747	.	.	.	-	
** - Significant at 1%, * - Significant  at 5%, NS - Non Significant	


Analysis Performed at IASRI Server


Block Design Experiment at NA For NA
ANOVA Analysis - Dependent Variable is _5s3

Source	DF	Type III SS	Mean Square	F Value	Pr > F	Significant	
Rep	2	186.7693	93.3846	4.0867	0.0344	*	
Trt	9	1074.7464	119.4163	5.2259	0.0014	**	
Error	18	411.3143	22.8508	.	.	-	
Corrected Total	29	1672.8300	.	.	.	-	
** - Significant at 1%, * - Significant  at 5%, NS - Non Significant	


Analysis Performed at IASRI Server


Block Design Experiment at NA For NA
ANOVA Analysis - Dependent Variable is _5s4

Source	DF	Type III SS	Mean Square	F Value	Pr > F	Significant	
Rep	2	89.3888	44.6944	2.3614	0.1228	NS	
Trt	9	1392.8741	154.7638	8.1768	0.0001	**	
Error	18	340.6913	18.9273	.	.	-	
Corrected Total	29	1822.9542	.	.	.	-	
** - Significant at 1%, * - Significant  at 5%, NS - Non Significant	


Analysis Performed at IASRI Server


Block Design Experiment at NA For NA
ANOVA Analysis - Dependent Variable is _5s5

Source	DF	Type III SS	Mean Square	F Value	Pr > F	Significant	
Rep	2	84.4616	42.2308	1.3521	0.2837	NS	
Trt	9	820.3207	91.1467	2.9183	0.0254	*	
Error	18	562.1931	31.2330	.	.	-	
Corrected Total	29	1466.9754	.	.	.	-	
** - Significant at 1%, * - Significant  at 5%, NS - Non Significant	


Analysis Performed at IASRI Server


Block Design Experiment at NA For NA
ANOVA Analysis - Dependent Variable is _6s5

Source	DF	Type III SS	Mean Square	F Value	Pr > F	Significant	
Rep	2	9.3309	4.6654	0.1284	0.8803	NS	
Trt	9	1403.2807	155.9201	4.2901	0.0042	**	
Error	18	654.2014	36.3445	.	.	-	
Corrected Total	29	2066.8129	.	.	.	-	
** - Significant at 1%, * - Significant  at 5%, NS - Non Significant	


Analysis Performed at IASRI Server


Block Design Experiment at NA For NA
ANOVA Analysis - Dependent Variable is _7s5

Source	DF	Type III SS	Mean Square	F Value	Pr > F	Significant	
Rep	2	112.1801	56.0900	1.2513	0.3099	NS	
Trt	9	1463.5541	162.6171	3.6279	0.0096	**	
Error	18	806.8304	44.8239	.	.	-	
Corrected Total	29	2382.5646	.	.	.	-	
** - Significant at 1%, * - Significant  at 5%, NS - Non Significant	


Analysis Performed at IASRI Server


Block Design Experiment at NA For NA
ANOVA Analysis - Dependent Variable is _8s5

Source	DF	Type III SS	Mean Square	F Value	Pr > F	Significant	
Rep	2	61.4811	30.7405	0.6236	0.5472	NS	
Trt	9	1594.6631	177.1848	3.5942	0.0100	**	
Error	18	887.3654	49.2981	.	.	-	
Corrected Total	29	2543.5095	.	.	.	-	
** - Significant at 1%, * - Significant  at 5%, NS - Non Significant	


Analysis Performed at IASRI Server


Block Design Experiment at NA For NA
ANOVA Analysis - Dependent Variable is _9s5

Source	DF	Type III SS	Mean Square	F Value	Pr > F	Significant	
Rep	2	121.9632	60.9816	1.1795	0.3301	NS	
Trt	9	2499.1149	277.6794	5.3707	0.0012	**	
Error	18	930.6413	51.7023	.	.	-	
Corrected Total	29	3551.7193	.	.	.	-	
** - Significant at 1%, * - Significant  at 5%, NS - Non Significant	


Analysis Performed at IASRI Server


Block Design Experiment at NA For NA
Treatment Mean Table

Trt	Mgt	Sgi	Svi_i	
Treatment Name	Treatment Description	Treatment of Mgt	Rank of Treatment	Treatment of Sgi	Rank of Treatment	Treatment of Svi_i	Rank of Treatment	
1		16.17	2	4.87	8	2.71	7	
2		14.85	5	5.80	6	3.47	5	
3		16.85	1	4.01	10	2.14	10	
4		13.29	9	6.92	2	3.80	3	
5		12.25	10	7.30	1	4.08	2	
6		14.42	6	4.90	7	2.26	8	
7		13.51	8	4.46	9	2.23	9	
8		14.18	7	5.97	5	4.34	1	
9		14.90	4	6.07	3	3.48	4	
10		15.74	3	6.01	4	3.36	6	
General Mean		14.62	.	5.63	.	3.19	.	
p-Value		0.3645	.	0.0012	.	0.0025	.	
CV(%)		15.26	.	13.98	.	20.02	.	
SE(d)		1.821	.	0.643	.	0.521	.	
LSD at 5%		NS	.	1.3506	.	1.0944	.	

Svi__ii	_10s5	_1s0	_1s1	_1s2	
Treatment of Svi__ii	Rank of Treatment	Treatment of _10s5	Rank of Treatment	Treatment of _1s0	Rank of Treatment	Treatment of _1s1	Rank of Treatment	Treatment of _1s2	Rank of Treatment	
7.12	8	0.00	9	57.91	2	25.31	9	18.05	10	
8.80	6	14.76	7	42.13	8	34.15	6	28.67	2	
8.29	7	31.78	2	58.07	1	21.34	10	21.90	9	
11.37	4	18.05	3	45.03	5	34.18	3	25.00	4	
13.16	2	15.00	6	42.12	9	34.18	4	28.86	1	
6.33	9	-0.00	10	47.88	4	33.16	7	22.60	8	
5.86	10	12.29	8	53.76	3	25.31	8	23.86	7	
15.12	1	32.09	1	45.00	6	34.18	5	25.00	5	
11.53	3	16.60	5	44.03	7	36.24	2	24.05	6	
10.55	5	16.60	4	42.12	10	37.26	1	25.19	3	
9.81	.	15.72	.	47.81	.	31.53	.	24.32	.	
0.0053	.	<.0001	.	0.0003	.	<.0001	.	0.1974	.	
26.56	.	41.13	.	9.01	.	7.32	.	17.92	.	
2.128	.	5.278	.	3.517	.	1.884	.	3.557	.	
4.4704	.	11.088	.	7.3886	.	3.9581	.	NS	.	

_2s0	_2s1	_2s2	_2s3	_3s0	
Treatment of _2s0	Rank of Treatment	Treatment of _2s1	Rank of Treatment	Treatment of _2s2	Rank of Treatment	Treatment of _2s3	Rank of Treatment	Treatment of _3s0	Rank of Treatment	
36.24	3	25.00	6	36.24	1	19.50	9	31.07	4	
33.16	5	34.15	2	35.25	2	10.45	10	24.31	7	
48.93	1	18.61	9	17.60	10	24.31	8	46.05	1	
25.31	9	23.53	7	28.54	4	45.96	1	22.60	9	
24.05	10	14.76	10	30.95	3	44.99	2	22.79	8	
36.24	4	28.86	4	24.05	7	29.93	5	34.18	3	
44.04	2	21.34	8	21.34	8	29.93	6	41.15	2	
27.71	8	29.93	3	21.34	9	39.21	3	26.45	5	
32.14	6	25.31	5	25.31	5	36.24	4	26.45	6	
28.86	7	36.24	1	24.05	6	29.93	7	22.60	10	
33.67	.	25.77	.	26.46	.	31.04	.	29.76	.	
<.0001	.	0.0162	.	0.0193	.	<.0001	.	0.0002	.	
9.95	.	25.04	.	22.85	.	19.95	.	17.71	.	
2.736	.	5.268	.	4.938	.	5.057	.	4.304	.	
5.7472	.	11.068	.	10.375	.	10.625	.	9.0425	.	

_3s1	_3s2	_3s3	_3s4	_4s0	
Treatment of _3s1	Rank of Treatment	Treatment of _3s2	Rank of Treatment	Treatment of _3s3	Rank of Treatment	Treatment of _3s4	Rank of Treatment	Treatment of _4s0	Rank of Treatment	
23.86	2	12.92	5	39.21	3	19.50	10	30.00	4	
24.05	1	18.05	4	28.86	7	34.81	2	23.16	7	
17.47	5	10.45	7	23.16	10	23.16	9	43.08	1	
16.60	7	20.26	3	30.95	6	32.30	3	22.60	10	
12.92	10	8.61	10	34.18	5	42.12	1	22.79	8	
18.05	3	26.45	1	25.31	9	26.45	7	34.18	3	
16.21	8	10.45	8	28.78	8	26.45	8	41.15	2	
18.05	4	10.45	9	41.15	2	27.60	6	26.45	6	
14.76	9	21.34	2	36.24	4	29.93	5	26.45	5	
16.60	6	10.45	6	42.12	1	31.07	4	22.60	9	
17.85	.	14.94	.	33.00	.	29.34	.	29.25	.	
0.1231	.	0.1590	.	0.0003	.	0.0451	.	0.0003	.	
25.27	.	54.16	.	13.19	.	23.50	.	17.74	.	
3.684	.	6.609	.	3.555	.	5.630	.	4.235	.	
NS	.	NS	.	7.4682	.	11.829	.	8.8981	.	

_4s1	_4s2	_4s3	_4s4	_4s5	
Treatment of _4s1	Rank of Treatment	Treatment of _4s2	Rank of Treatment	Treatment of _4s3	Rank of Treatment	Treatment of _4s4	Rank of Treatment	Treatment of _4s5	Rank of Treatment	
18.05	2	24.98	1	30.95	1	33.16	9	0.00	5	
22.79	1	12.92	3	25.31	6	41.12	6	0.00	6	
12.92	7	4.31	7	21.34	8	35.68	8	6.14	1	
6.14	9	0.00	10	28.67	3	49.80	2	0.00	7	
12.92	8	4.31	8	27.22	4	48.87	3	0.00	8	
16.21	3	22.60	2	22.60	7	33.04	10	0.00	9	
14.76	5	4.31	9	20.76	9	36.24	7	0.00	10	
13.74	6	6.14	6	16.60	10	50.79	1	6.14	2	
6.14	10	10.45	4	29.93	2	43.08	5	0.00	4	
14.76	4	6.14	5	26.45	5	47.88	4	0.00	3	
13.84	.	9.62	.	24.98	.	41.97	.	1.23	.	
0.2061	.	0.0270	.	0.0280	.	0.0011	.	0.5887	.	
50.33	.	87.95	.	18.77	.	12.54	.	397.91	.	
5.689	.	6.906	.	3.829	.	4.298	.	3.993	.	
NS	.	14.508	.	8.0436	.	9.0298	.	NS	.	

_5s0	_5s1	_5s2	_5s3	_5s4	
Treatment of _5s0	Rank of Treatment	Treatment of _5s1	Rank of Treatment	Treatment of _5s2	Rank of Treatment	Treatment of _5s3	Rank of Treatment	Treatment of _5s4	Rank of Treatment	
27.71	4	19.50	2	10.45	2	27.71	1	39.21	8	
23.16	7	22.79	1	4.31	5	22.79	4	46.00	6	
43.08	1	12.92	7	4.31	6	10.45	9	40.11	7	
22.60	9	6.14	9	0.00	9	26.26	2	51.81	3	
22.79	8	12.92	8	4.31	8	21.14	7	53.76	2	
34.18	3	16.21	3	16.60	1	22.60	6	37.12	10	
41.15	2	14.76	5	6.14	3	18.05	8	37.26	9	
26.45	5	13.74	6	4.31	7	8.61	10	54.89	1	
26.45	6	6.14	10	0.00	10	23.74	3	50.79	5	
22.60	10	14.76	4	4.31	4	22.60	5	51.76	4	
29.02	.	13.99	.	5.47	.	20.39	.	46.27	.	
0.0004	.	0.1886	.	0.1033	.	0.0014	.	<.0001	.	
18.15	.	50.44	.	109.99	.	23.44	.	9.40	.	
4.300	.	5.761	.	4.915	.	3.903	.	3.552	.	
9.035	.	NS	.	NS	.	8.2	.	7.4629	.	

_5s5	_6s5	_7s5	_8s5	_9s5	
Treatment of _5s5	Rank of Treatment	Treatment of _6s5	Rank of Treatment	Treatment of _7s5	Rank of Treatment	Treatment of _8s5	Rank of Treatment	Treatment of _9s5	Rank of Treatment	
0.00	9	0.00	7	-0.00	10	0.00	9	0.00	9	
0.00	6	4.31	4	4.31	6	4.31	8	12.92	6	
8.86	2	19.31	1	20.76	1	22.02	2	29.31	1	
4.31	3	4.31	5	8.61	3	10.45	3	18.05	3	
0.00	7	0.00	8	4.31	7	8.61	5	13.74	5	
0.00	10	0.00	9	0.00	9	0.00	10	-0.00	10	
0.00	8	8.61	3	8.61	4	8.61	6	12.29	7	
16.21	1	17.47	2	20.45	2	22.29	1	27.52	2	
0.00	4	0.00	10	4.31	8	8.61	4	12.29	8	
0.00	5	4.31	6	8.61	5	8.61	7	14.76	4	
2.94	.	5.83	.	8.00	.	9.35	.	14.09	.	
0.0254	.	0.0042	.	0.0096	.	0.0100	.	0.0012	.	
190.27	.	103.39	.	83.72	.	75.08	.	51.04	.	
4.563	.	4.923	.	5.467	.	5.733	.	5.871	.	
9.5867	.	10.342	.	11.485	.	12.044	.	12.334	.	


Means with atleast one letter common are not statistically significant using
Fisher's Least Significant Difference


Analysis Performed at IASRI Server


Block Design Experiment at NA For NA
Treatment Details Table

Obs	Treatment Name	Treatment Details	
1	1		
2	2		
3	3		
4	4		
5	5		
6	6		
7	7		
8	8		
9	9		
10	10		


Analysis Performed at IASRI Server
