# Supplementary material for: Data on germination, growth and morphological changes of oil palm (Elaeis guineensis Jacq.) zygotic embryos during in vitro culturing
Source: Data Brief. 2019 Dec 16;28:104975. doi: 10.1016/j.dib.2019.104975 (PMC7093796; doi:10.1016/j.dib.2019.104975)
Supplement: Multimedia component 7 [file mmc7.zip › dib_104975_G2 Block_sscnars49 ger GL_V2_mmc7.RTF]

CENTER: NA
Experiment No: NACrop: NA
Year: NASeason: NA
Block Design Experiment at NA For NA
ANOVA Analysis - Dependent Variable is MGT

Source	DF	Type III SS	Mean Square	F Value	Pr > F	Significant	
Rep	2	10.3011	5.1506	11.2290	0.0007	**	
Trt	9	73.3666	8.1518	17.7723	<.0001	**	
Error	18	8.2563	0.4587	.	.	-	
Corrected Total	29	91.9240	.	.	.	-	
** - Significant at 1%, * - Significant  at 5%, NS - Non Significant	


Analysis Performed at IASRI Server


Block Design Experiment at NA For NA
ANOVA Analysis - Dependent Variable is SGI

Source	DF	Type III SS	Mean Square	F Value	Pr > F	Significant	
Rep	2	12.4312	6.2156	6.1579	0.0092	**	
Trt	9	20.6429	2.2937	2.2724	0.0661	NS	
Error	18	18.1687	1.0094	.	.	-	
Corrected Total	29	51.2428	.	.	.	-	
** - Significant at 1%, * - Significant  at 5%, NS - Non Significant	


Analysis Performed at IASRI Server


Block Design Experiment at NA For NA
ANOVA Analysis - Dependent Variable is SVI__1

Source	DF	Type III SS	Mean Square	F Value	Pr > F	Significant	
Rep	2	3.1422	1.5711	5.2605	0.0159	*	
Trt	9	2.7611	0.3068	1.0272	0.4560	NS	
Error	18	5.3759	0.2987	.	.	-	
Corrected Total	29	11.2792	.	.	.	-	
** - Significant at 1%, * - Significant  at 5%, NS - Non Significant	


Analysis Performed at IASRI Server


Block Design Experiment at NA For NA
ANOVA Analysis - Dependent Variable is VAR35

Source	DF	Type III SS	Mean Square	F Value	Pr > F	Significant	
Rep	2	15.3646	7.6823	1.7383	0.2041	NS	
Trt	9	70.8440	7.8716	1.7812	0.1421	NS	
Error	18	79.5484	4.4194	.	.	-	
Corrected Total	29	165.7569	.	.	.	-	
** - Significant at 1%, * - Significant  at 5%, NS - Non Significant	


Analysis Performed at IASRI Server


Block Design Experiment at NA For NA
ANOVA Analysis - Dependent Variable is _10s5

Source	DF	Type III SS	Mean Square	F Value	Pr > F	Significant	
Rep	2	130.1029	65.0515	1.4245	0.2665	NS	
Trt	9	2905.1086	322.7898	7.0682	0.0002	**	
Error	18	822.0170	45.6676	.	.	-	
Corrected Total	29	3857.2285	.	.	.	-	
** - Significant at 1%, * - Significant  at 5%, NS - Non Significant	


Analysis Performed at IASRI Server


Block Design Experiment at NA For NA
ANOVA Analysis - Dependent Variable is _1s0

Source	DF	Type III SS	Mean Square	F Value	Pr > F	Significant	
Rep	2	224.3797	112.1899	4.5469	0.0252	*	
Trt	9	935.0741	103.8971	4.2108	0.0046	**	
Error	18	444.1340	24.6741	.	.	-	
Corrected Total	29	1603.5879	.	.	.	-	
** - Significant at 1%, * - Significant  at 5%, NS - Non Significant	


Analysis Performed at IASRI Server


Block Design Experiment at NA For NA
ANOVA Analysis - Dependent Variable is _1s1

Source	DF	Type III SS	Mean Square	F Value	Pr > F	Significant	
Rep	2	126.7208	63.3604	3.3984	0.0560	NS	
Trt	9	407.2612	45.2512	2.4271	0.0522	NS	
Error	18	335.5994	18.6444	.	.	-	
Corrected Total	29	869.5814	.	.	.	-	
** - Significant at 1%, * - Significant  at 5%, NS - Non Significant	


Analysis Performed at IASRI Server


Block Design Experiment at NA For NA
ANOVA Analysis - Dependent Variable is _1s2

Source	DF	Type III SS	Mean Square	F Value	Pr > F	Significant	
Rep	2	99.3459	49.6729	3.7191	0.0445	*	
Trt	9	746.7642	82.9738	6.2123	0.0005	**	
Error	18	240.4142	13.3563	.	.	-	
Corrected Total	29	1086.5243	.	.	.	-	
** - Significant at 1%, * - Significant  at 5%, NS - Non Significant	


Analysis Performed at IASRI Server


Block Design Experiment at NA For NA
ANOVA Analysis - Dependent Variable is _2s0

Source	DF	Type III SS	Mean Square	F Value	Pr > F	Significant	
Rep	2	392.6922	196.3461	4.4030	0.0278	*	
Trt	9	1316.2557	146.2506	3.2796	0.0153	*	
Error	18	802.6861	44.5937	.	.	-	
Corrected Total	29	2511.6341	.	.	.	-	
** - Significant at 1%, * - Significant  at 5%, NS - Non Significant	


Analysis Performed at IASRI Server


Block Design Experiment at NA For NA
ANOVA Analysis - Dependent Variable is _2s1

Source	DF	Type III SS	Mean Square	F Value	Pr > F	Significant	
Rep	2	21.0061	10.5031	0.6206	0.5487	NS	
Trt	9	342.4654	38.0517	2.2483	0.0686	NS	
Error	18	304.6453	16.9247	.	.	-	
Corrected Total	29	668.1169	.	.	.	-	
** - Significant at 1%, * - Significant  at 5%, NS - Non Significant	


Analysis Performed at IASRI Server


Block Design Experiment at NA For NA
ANOVA Analysis - Dependent Variable is _2s2

Source	DF	Type III SS	Mean Square	F Value	Pr > F	Significant	
Rep	2	183.1215	91.5608	4.1352	0.0333	*	
Trt	9	854.7976	94.9775	4.2895	0.0042	**	
Error	18	398.5564	22.1420	.	.	-	
Corrected Total	29	1436.4755	.	.	.	-	
** - Significant at 1%, * - Significant  at 5%, NS - Non Significant	


Analysis Performed at IASRI Server


Block Design Experiment at NA For NA
ANOVA Analysis - Dependent Variable is _2s3

Source	DF	Type III SS	Mean Square	F Value	Pr > F	Significant	
Rep	2	560.2465	280.1232	7.0544	0.0055	**	
Trt	9	2379.8551	264.4283	6.6592	0.0003	**	
Error	18	714.7597	39.7089	.	.	-	
Corrected Total	29	3654.8613	.	.	.	-	
** - Significant at 1%, * - Significant  at 5%, NS - Non Significant	


Analysis Performed at IASRI Server


Block Design Experiment at NA For NA
ANOVA Analysis - Dependent Variable is _3s0

Source	DF	Type III SS	Mean Square	F Value	Pr > F	Significant	
Rep	2	812.3478	406.1739	5.9640	0.0103	*	
Trt	9	601.5761	66.8418	0.9815	0.4868	NS	
Error	18	1225.8721	68.1040	.	.	-	
Corrected Total	29	2639.7960	.	.	.	-	
** - Significant at 1%, * - Significant  at 5%, NS - Non Significant	


Analysis Performed at IASRI Server


Block Design Experiment at NA For NA
ANOVA Analysis - Dependent Variable is _3s1

Source	DF	Type III SS	Mean Square	F Value	Pr > F	Significant	
Rep	2	22.2587	11.1294	0.6321	0.5429	NS	
Trt	9	665.5648	73.9516	4.2001	0.0046	**	
Error	18	316.9289	17.6072	.	.	-	
Corrected Total	29	1004.7524	.	.	.	-	
** - Significant at 1%, * - Significant  at 5%, NS - Non Significant	


Analysis Performed at IASRI Server


Block Design Experiment at NA For NA
ANOVA Analysis - Dependent Variable is _3s2

Source	DF	Type III SS	Mean Square	F Value	Pr > F	Significant	
Rep	2	784.8715	392.4358	8.2780	0.0028	**	
Trt	9	1199.0028	133.2225	2.8102	0.0297	*	
Error	18	853.3248	47.4069	.	.	-	
Corrected Total	29	2837.1991	.	.	.	-	
** - Significant at 1%, * - Significant  at 5%, NS - Non Significant	


Analysis Performed at IASRI Server


Block Design Experiment at NA For NA
ANOVA Analysis - Dependent Variable is _3s3

Source	DF	Type III SS	Mean Square	F Value	Pr > F	Significant	
Rep	2	1506.6093	753.3047	8.6934	0.0023	**	
Trt	9	703.1605	78.1289	0.9016	0.5438	NS	
Error	18	1559.7497	86.6528	.	.	-	
Corrected Total	29	3769.5196	.	.	.	-	
** - Significant at 1%, * - Significant  at 5%, NS - Non Significant	


Analysis Performed at IASRI Server


Block Design Experiment at NA For NA
ANOVA Analysis - Dependent Variable is _3s4

Source	DF	Type III SS	Mean Square	F Value	Pr > F	Significant	
Rep	2	115.7850	57.8925	2.2261	0.1368	NS	
Trt	9	1048.6247	116.5139	4.4803	0.0033	**	
Error	18	468.1092	26.0061	.	.	-	
Corrected Total	29	1632.5189	.	.	.	-	
** - Significant at 1%, * - Significant  at 5%, NS - Non Significant	


Analysis Performed at IASRI Server


Block Design Experiment at NA For NA
ANOVA Analysis - Dependent Variable is _4s0

Source	DF	Type III SS	Mean Square	F Value	Pr > F	Significant	
Rep	2	706.6194	353.3097	4.5251	0.0256	*	
Trt	9	820.8104	91.2012	1.1681	0.3704	NS	
Error	18	1405.4098	78.0783	.	.	-	
Corrected Total	29	2932.8396	.	.	.	-	
** - Significant at 1%, * - Significant  at 5%, NS - Non Significant	


Analysis Performed at IASRI Server


Block Design Experiment at NA For NA
ANOVA Analysis - Dependent Variable is _4s1

Source	DF	Type III SS	Mean Square	F Value	Pr > F	Significant	
Rep	2	58.5537	29.2768	0.7599	0.4821	NS	
Trt	9	531.9389	59.1043	1.5341	0.2099	NS	
Error	18	693.4757	38.5264	.	.	-	
Corrected Total	29	1283.9683	.	.	.	-	
** - Significant at 1%, * - Significant  at 5%, NS - Non Significant	


Analysis Performed at IASRI Server


Block Design Experiment at NA For NA
ANOVA Analysis - Dependent Variable is _4s2

Source	DF	Type III SS	Mean Square	F Value	Pr > F	Significant	
Rep	2	0.2389	0.1194	0.0023	0.9977	NS	
Trt	9	683.0467	75.8941	1.4616	0.2353	NS	
Error	18	934.6702	51.9261	.	.	-	
Corrected Total	29	1617.9558	.	.	.	-	
** - Significant at 1%, * - Significant  at 5%, NS - Non Significant	


Analysis Performed at IASRI Server


Block Design Experiment at NA For NA
ANOVA Analysis - Dependent Variable is _4s3

Source	DF	Type III SS	Mean Square	F Value	Pr > F	Significant	
Rep	2	43.5923	21.7962	0.9635	0.4004	NS	
Trt	9	484.3596	53.8177	2.3791	0.0562	NS	
Error	18	407.1724	22.6207	.	.	-	
Corrected Total	29	935.1243	.	.	.	-	
** - Significant at 1%, * - Significant  at 5%, NS - Non Significant	


Analysis Performed at IASRI Server


Block Design Experiment at NA For NA
ANOVA Analysis - Dependent Variable is _4s4

Source	DF	Type III SS	Mean Square	F Value	Pr > F	Significant	
Rep	2	356.2581	178.1291	3.9508	0.0378	*	
Trt	9	1218.0804	135.3423	3.0018	0.0225	*	
Error	18	811.5572	45.0865	.	.	-	
Corrected Total	29	2385.8958	.	.	.	-	
** - Significant at 1%, * - Significant  at 5%, NS - Non Significant	


Analysis Performed at IASRI Server


Block Design Experiment at NA For NA
ANOVA Analysis - Dependent Variable is _4s5

Source	DF	Type III SS	Mean Square	F Value	Pr > F	Significant	
Rep	2	8.4073	4.2036	0.3227	0.7283	NS	
Trt	9	747.0003	83.0000	6.3720	0.0004	**	
Error	18	234.4639	13.0258	.	.	-	
Corrected Total	29	989.8715	.	.	.	-	
** - Significant at 1%, * - Significant  at 5%, NS - Non Significant	


Analysis Performed at IASRI Server


Block Design Experiment at NA For NA
ANOVA Analysis - Dependent Variable is _5s0

Source	DF	Type III SS	Mean Square	F Value	Pr > F	Significant	
Rep	2	706.6194	353.3097	4.5251	0.0256	*	
Trt	9	820.8104	91.2012	1.1681	0.3704	NS	
Error	18	1405.4098	78.0783	.	.	-	
Corrected Total	29	2932.8396	.	.	.	-	
** - Significant at 1%, * - Significant  at 5%, NS - Non Significant	


Analysis Performed at IASRI Server


Block Design Experiment at NA For NA
ANOVA Analysis - Dependent Variable is _5s1

Source	DF	Type III SS	Mean Square	F Value	Pr > F	Significant	
Rep	2	58.5537	29.2768	0.7599	0.4821	NS	
Trt	9	531.9389	59.1043	1.5341	0.2099	NS	
Error	18	693.4757	38.5264	.	.	-	
Corrected Total	29	1283.9683	.	.	.	-	
** - Significant at 1%, * - Significant  at 5%, NS - Non Significant	


Analysis Performed at IASRI Server


Block Design Experiment at NA For NA
ANOVA Analysis - Dependent Variable is _5s2

Source	DF	Type III SS	Mean Square	F Value	Pr > F	Significant	
Rep	2	11.1301	5.5650	0.1304	0.8785	NS	
Trt	9	383.9882	42.6654	1.0000	0.4742	NS	
Error	18	767.9763	42.6654	.	.	-	
Corrected Total	29	1163.0946	.	.	.	-	
** - Significant at 1%, * - Significant  at 5%, NS - Non Significant	


Analysis Performed at IASRI Server


Block Design Experiment at NA For NA
ANOVA Analysis - Dependent Variable is _5s3

Source	DF	Type III SS	Mean Square	F Value	Pr > F	Significant	
Rep	2	75.4018	37.7009	1.7347	0.2047	NS	
Trt	9	932.3812	103.5979	4.7667	0.0024	**	
Error	18	391.2098	21.7339	.	.	-	
Corrected Total	29	1398.9929	.	.	.	-	
** - Significant at 1%, * - Significant  at 5%, NS - Non Significant	


Analysis Performed at IASRI Server


Block Design Experiment at NA For NA
ANOVA Analysis - Dependent Variable is _5s4

Source	DF	Type III SS	Mean Square	F Value	Pr > F	Significant	
Rep	2	375.8912	187.9456	3.2140	0.0640	NS	
Trt	9	1065.2849	118.3650	2.0241	0.0970	NS	
Error	18	1052.5848	58.4769	.	.	-	
Corrected Total	29	2493.7609	.	.	.	-	
** - Significant at 1%, * - Significant  at 5%, NS - Non Significant	


Analysis Performed at IASRI Server


Block Design Experiment at NA For NA
ANOVA Analysis - Dependent Variable is _5s5

Source	DF	Type III SS	Mean Square	F Value	Pr > F	Significant	
Rep	2	1.2624	0.6312	0.0486	0.9527	NS	
Trt	9	1291.8631	143.5403	11.0433	<.0001	**	
Error	18	233.9634	12.9980	.	.	-	
Corrected Total	29	1527.0889	.	.	.	-	
** - Significant at 1%, * - Significant  at 5%, NS - Non Significant	


Analysis Performed at IASRI Server


Block Design Experiment at NA For NA
ANOVA Analysis - Dependent Variable is _6s5

Source	DF	Type III SS	Mean Square	F Value	Pr > F	Significant	
Rep	2	165.7665	82.8833	2.4745	0.1124	NS	
Trt	9	1273.4207	141.4912	4.2243	0.0045	**	
Error	18	602.9096	33.4950	.	.	-	
Corrected Total	29	2042.0969	.	.	.	-	
** - Significant at 1%, * - Significant  at 5%, NS - Non Significant	


Analysis Performed at IASRI Server


Block Design Experiment at NA For NA
ANOVA Analysis - Dependent Variable is _7s5

Source	DF	Type III SS	Mean Square	F Value	Pr > F	Significant	
Rep	2	69.4996	34.7498	0.8634	0.4385	NS	
Trt	9	1484.3903	164.9323	4.0978	0.0053	**	
Error	18	724.4804	40.2489	.	.	-	
Corrected Total	29	2278.3703	.	.	.	-	
** - Significant at 1%, * - Significant  at 5%, NS - Non Significant	


Analysis Performed at IASRI Server


Block Design Experiment at NA For NA
ANOVA Analysis - Dependent Variable is _8s5

Source	DF	Type III SS	Mean Square	F Value	Pr > F	Significant	
Rep	2	85.5898	42.7949	1.0874	0.3582	NS	
Trt	9	1601.3892	177.9321	4.5212	0.0032	**	
Error	18	708.3902	39.3550	.	.	-	
Corrected Total	29	2395.3692	.	.	.	-	
** - Significant at 1%, * - Significant  at 5%, NS - Non Significant	


Analysis Performed at IASRI Server


Block Design Experiment at NA For NA
ANOVA Analysis - Dependent Variable is _9s5

Source	DF	Type III SS	Mean Square	F Value	Pr > F	Significant	
Rep	2	133.7033	66.8516	1.4816	0.2537	NS	
Trt	9	2490.7257	276.7473	6.1336	0.0006	**	
Error	18	812.1592	45.1200	.	.	-	
Corrected Total	29	3436.5881	.	.	.	-	
** - Significant at 1%, * - Significant  at 5%, NS - Non Significant	


Analysis Performed at IASRI Server


Block Design Experiment at NA For NA
Treatment Mean Table

Trt	Mgt	Sgi	Svi__1	
Treatment Name	Treatment Description	Treatment of Mgt	Rank of Treatment	Treatment of Sgi	Rank of Treatment	Treatment of Svi__1	Rank of Treatment	
1		16.87ABC               	3	4.52	9	2.13	9	
2		16.39BC                	5	4.88	7	2.30	7	
3		15.97C                 	6	5.36	6	3.01	1	
4		17.26AB                	2	4.87	8	2.31	6	
5		16.44BC                	4	5.67	4	2.52	3	
6		13.10D                 	10	6.05	3	2.18	8	
7		17.65A                 	1	4.31	10	1.96	10	
8		13.85D                 	9	5.60	5	2.51	5	
9		14.00D                 	7	6.55	2	2.52	4	
10		13.99D                 	8	7.00	1	2.83	2	
General Mean		15.55	.	5.48	.	2.43	.	
p-Value		<.0001	.	0.0661	.	0.4560	.	
CV(%)		4.35	.	18.33	.	22.53	.	
SE(d)		0.553	.	0.820	.	0.446	.	
LSD at 5%		1.1618	.	NS	.	NS	.	

Var35	_10s5	_1s0	_1s1	_1s2	
Treatment of Var35	Rank of Treatment	Treatment of _10s5	Rank of Treatment	Treatment of _1s0	Rank of Treatment	Treatment of _1s1	Rank of Treatment	Treatment of _1s2	Rank of Treatment	
6.18	8	0.00C                 	9	50.79A                 	3	33.21	7	18.05C                 	9	
7.09	7	12.29B                 	6	50.96A                 	2	34.02	5	16.60C                 	10	
9.89	2	32.02A                 	1	47.96AB                	5	34.04	4	20.76C                 	7	
7.42	6	8.61BC                	8	47.91AB                	6	35.22	3	19.50C                 	8	
8.68	5	14.76B                 	3	48.08AB                	4	33.73	6	21.34C                 	6	
6.18	9	0.00C                 	10	42.12BC                	8	33.16	8	29.93A                 	2	
5.98	10	11.90B                 	7	52.74A                 	1	27.71	10	22.79BC                	5	
10.06	1	28.67A                 	2	45.97AB                	7	29.93	9	28.67AB                	3	
9.30	4	12.29B                 	5	37.26C                 	9	37.26	2	31.07A                 	1	
9.34	3	12.92B                 	4	35.25C                 	10	42.13	1	27.71AB                	4	
8.01	.	13.35	.	45.90	.	34.04	.	23.64	.	
0.1421	.	0.0002	.	0.0046	.	0.0522	.	0.0005	.	
26.24	.	50.64	.	10.82	.	12.68	.	15.46	.	
1.716	.	5.518	.	4.056	.	3.526	.	2.984	.	
NS	.	11.592	.	8.5209	.	NS	.	6.2691	.	

_2s0	_2s1	_2s2	_2s3	_3s0	
Treatment of _2s0	Rank of Treatment	Treatment of _2s1	Rank of Treatment	Treatment of _2s2	Rank of Treatment	Treatment of _2s3	Rank of Treatment	Treatment of _3s0	Rank of Treatment	
41.13A                 	2	29.93	4	24.05D                 	10	22.60ABC               	4	32.30	2	
37.87A                 	5	26.07	9	33.16BC                	4	19.31BCD               	5	30.19	5	
39.01A                 	4	25.00	10	32.76BC                	5	16.45CD                	8	26.41	7	
39.04A                 	3	32.02	3	28.24BCD               	7	16.60CD                	7	31.45	4	
36.61A                 	6	26.57	7	31.75BCD               	6	18.86BCD               	6	24.98	8	
33.16ABC               	8	26.45	8	35.25AB                	2	24.05ABC               	3	27.71	6	
44.04A                 	1	34.18	2	25.31CD                	9	-0.00E                 	10	32.09	3	
35.17AB                	7	28.86	5	27.71BCD               	8	27.60AB                	2	33.16	1	
23.86BC                	9	35.25	1	43.09A                 	1	8.61DE                	9	21.34	9	
22.79C                 	10	27.71	6	35.17AB                	3	33.16A                 	1	19.89	10	
35.27	.	29.20	.	31.65	.	18.72	.	27.95	.	
0.0153	.	0.0686	.	0.0042	.	0.0003	.	0.4868	.	
18.93	.	14.09	.	14.87	.	33.66	.	29.52	.	
5.452	.	3.359	.	3.842	.	5.145	.	6.738	.	
11.455	.	NS	.	8.0718	.	10.81	.	NS	.	

_3s1	_3s2	_3s3	_3s4	_4s0	
Treatment of _3s1	Rank of Treatment	Treatment of _3s2	Rank of Treatment	Treatment of _3s3	Rank of Treatment	Treatment of _3s4	Rank of Treatment	Treatment of _4s0	Rank of Treatment	
16.60BCD               	5	24.23A                 	1	37.60	8	12.92D                 	10	31.31	3	
14.76CD                	7	15.00ABC               	7	40.81	6	20.76BCD               	6	30.19	4	
12.92CD                	9	18.61AB                	4	38.10	7	25.25AB                	3	22.10	6	
10.45D                 	10	18.61AB                	5	44.43	3	12.92D                 	9	21.52	7	
12.92CD                	8	22.54A                 	2	41.53	4	20.76BCD               	7	19.50	10	
22.60AB                	3	8.61BC                	8	36.24	9	29.93A                 	1	27.71	5	
23.86A                 	2	21.34A                 	3	31.07	10	22.60ABC               	4	32.09	2	
19.89ABC               	4	16.60AB                	6	41.15	5	14.76CD                	8	33.16	1	
24.05A                 	1	4.31C                 	10	47.88	1	21.34ABCD              	5	21.34	8	
14.76CD                	6	8.61BC                	9	46.95	2	29.93A                 	2	19.89	9	
17.28	.	15.85	.	40.58	.	21.12	.	25.88	.	
0.0046	.	0.0297	.	0.5438	.	0.0033	.	0.3704	.	
24.28	.	43.45	.	22.94	.	24.15	.	34.14	.	
3.426	.	5.622	.	7.600	.	4.164	.	7.215	.	
7.198	.	11.811	.	NS	.	8.7479	.	NS	.	

_4s1	_4s2	_4s3	_4s4	_4s5	
Treatment of _4s1	Rank of Treatment	Treatment of _4s2	Rank of Treatment	Treatment of _4s3	Rank of Treatment	Treatment of _4s4	Rank of Treatment	Treatment of _4s5	Rank of Treatment	
14.76	6	12.92	2	31.07	3	35.00BC                	9	-0.00B                 	9	
8.61	10	8.61	5	35.01	1	35.17BC                	8	4.31B                 	2	
14.76	7	4.31	7	23.86	8	46.92A                 	3	16.60A                 	1	
22.29	1	4.31	8	27.22	7	42.09AB                	5	0.00B                 	5	
14.31	8	10.45	3	23.74	9	48.93A                 	2	0.00B                 	6	
21.34	2	4.31	9	33.16	2	35.25BC                	7	-0.00B                 	10	
19.89	4	16.60	1	31.07	4	29.93C                 	10	0.00B                 	7	
16.60	5	10.45	4	22.60	10	40.20ABC               	6	4.31B                 	3	
21.34	3	4.31	6	29.93	5	43.09AB                	4	0.00B                 	8	
12.92	9	-0.00	10	29.93	6	49.80A                 	1	0.00B                 	4	
16.68	.	7.63	.	28.76	.	40.64	.	2.52	.	
0.2099	.	0.2353	.	0.0562	.	0.0225	.	0.0004	.	
37.21	.	94.49	.	16.54	.	16.52	.	143.16	.	
5.068	.	5.884	.	3.883	.	5.482	.	2.947	.	
NS	.	NS	.	NS	.	11.518	.	6.1911	.	

_5s0	_5s1	_5s2	_5s3	_5s4	
Treatment of _5s0	Rank of Treatment	Treatment of _5s1	Rank of Treatment	Treatment of _5s2	Rank of Treatment	Treatment of _5s3	Rank of Treatment	Treatment of _5s4	Rank of Treatment	
31.31	3	14.76	6	8.61	1	28.86AB                	2	38.05	9	
30.19	4	8.61	10	8.61	2	29.74A                 	1	40.11	7	
22.10	6	14.76	7	4.31	5	21.14BC                	8	48.93	3	
21.52	7	22.29	1	0.00	10	20.76C                 	9	48.90	4	
19.50	10	14.31	8	8.61	3	21.34BC                	7	52.11	2	
27.71	5	21.34	2	4.31	6	28.86AB                	3	39.21	8	
32.09	2	19.89	4	8.61	4	27.71ABC               	4	36.24	10	
33.16	1	16.60	5	0.00	9	10.45D                 	10	48.84	5	
21.34	8	21.34	3	4.31	7	26.45ABC               	5	45.96	6	
19.89	9	12.92	9	0.00	8	25.31ABC               	6	53.73	1	
25.88	.	16.68	.	4.74	.	24.06	.	45.21	.	
0.3704	.	0.2099	.	0.4742	.	0.0024	.	0.0970	.	
34.14	.	37.21	.	137.87	.	19.38	.	16.92	.	
7.215	.	5.068	.	5.333	.	3.806	.	6.244	.	
NS	.	NS	.	NS	.	7.9971	.	NS	.	

_5s5	_6s5	_7s5	_8s5	_9s5	
Treatment of _5s5	Rank of Treatment	Treatment of _6s5	Rank of Treatment	Treatment of _7s5	Rank of Treatment	Treatment of _8s5	Rank of Treatment	Treatment of _9s5	Rank of Treatment	
-0.00C                 	10	0.00C                 	10	-0.00C                 	10	0.00C                 	9	-0.00D                 	9	
8.61B                 	2	8.61BC                	4	10.45BC                	3	10.45BC                	3	12.29C                 	6	
21.34A                 	1	22.60A                 	1	24.05A                 	1	25.31A                 	1	31.00A                 	1	
0.00C                 	4	0.00C                 	8	4.31C                 	8	4.31C                 	8	8.61CD                	8	
0.00C                 	7	4.31BC                	5	8.61BC                	4	8.61BC                	4	14.76BC                	3	
0.00C                 	8	0.00C                 	9	0.00C                 	9	-0.00C                 	10	-0.00D                 	10	
0.00C                 	9	4.31BC                	6	8.61BC                	5	8.61BC                	5	11.90C                 	7	
4.31BC                	3	10.45B                 	2	18.05AB                	2	18.05AB                	2	25.00AB                	2	
0.00C                 	5	8.61BC                	3	8.61BC                	6	8.61BC                	6	12.29C                 	5	
0.00C                 	6	4.31BC                	7	8.61BC                	7	8.61BC                	7	12.92C                 	4	
3.43	.	6.32	.	9.13	.	9.26	.	12.88	.	
<.0001	.	0.0045	.	0.0053	.	0.0032	.	0.0006	.	
105.24	.	91.58	.	69.48	.	67.77	.	52.16	.	
2.944	.	4.725	.	5.180	.	5.122	.	5.485	.	
6.1845	.	9.9278	.	10.883	.	10.761	.	11.523	.	


Means with atleast one letter common are not statistically significant using
Fisher's Least Significant Difference

Grouping letters on treatments were made using pdglm800.sas which can be downloaded from http://animalscience.ag.utk.edu/FacultyStaff/ArnoldSaxton.html#software
Analysis Performed at IASRI Server


Block Design Experiment at NA For NA
Treatment Details Table

Obs	Treatment Name	Treatment Details	
1	1		
2	2		
3	3		
4	4		
5	5		
6	6		
7	7		
8	8		
9	9		
10	10		


Analysis Performed at IASRI Server
