# Supplementary material for: Data on germination, growth and morphological changes of oil palm (Elaeis guineensis Jacq.) zygotic embryos during in vitro culturing
Source: Data Brief. 2019 Dec 16;28:104975. doi: 10.1016/j.dib.2019.104975 (PMC7093796; doi:10.1016/j.dib.2019.104975)
Supplement: Multimedia component 8 [file mmc8.zip › dib_104975_G2 Block_sscnars49 ger_V2_mmc8.RTF]

CENTER: NA
Experiment No: NACrop: NA
Year: NASeason: NA
Block Design Experiment at NA For NA
ANOVA Analysis - Dependent Variable is MGT

Source	DF	Type III SS	Mean Square	F Value	Pr > F	Significant	
Rep	2	10.3011	5.1506	11.2290	0.0007	**	
Trt	9	73.3666	8.1518	17.7723	<.0001	**	
Error	18	8.2563	0.4587	.	.	-	
Corrected Total	29	91.9240	.	.	.	-	
** - Significant at 1%, * - Significant  at 5%, NS - Non Significant	


Analysis Performed at IASRI Server


Block Design Experiment at NA For NA
ANOVA Analysis - Dependent Variable is SGI

Source	DF	Type III SS	Mean Square	F Value	Pr > F	Significant	
Rep	2	12.4312	6.2156	6.1579	0.0092	**	
Trt	9	20.6429	2.2937	2.2724	0.0661	NS	
Error	18	18.1687	1.0094	.	.	-	
Corrected Total	29	51.2428	.	.	.	-	
** - Significant at 1%, * - Significant  at 5%, NS - Non Significant	


Analysis Performed at IASRI Server


Block Design Experiment at NA For NA
ANOVA Analysis - Dependent Variable is SVI__1

Source	DF	Type III SS	Mean Square	F Value	Pr > F	Significant	
Rep	2	3.1422	1.5711	5.2605	0.0159	*	
Trt	9	2.7611	0.3068	1.0272	0.4560	NS	
Error	18	5.3759	0.2987	.	.	-	
Corrected Total	29	11.2792	.	.	.	-	
** - Significant at 1%, * - Significant  at 5%, NS - Non Significant	


Analysis Performed at IASRI Server


Block Design Experiment at NA For NA
ANOVA Analysis - Dependent Variable is VAR35

Source	DF	Type III SS	Mean Square	F Value	Pr > F	Significant	
Rep	2	15.3646	7.6823	1.7383	0.2041	NS	
Trt	9	70.8440	7.8716	1.7812	0.1421	NS	
Error	18	79.5484	4.4194	.	.	-	
Corrected Total	29	165.7569	.	.	.	-	
** - Significant at 1%, * - Significant  at 5%, NS - Non Significant	


Analysis Performed at IASRI Server


Block Design Experiment at NA For NA
ANOVA Analysis - Dependent Variable is _10s5

Source	DF	Type III SS	Mean Square	F Value	Pr > F	Significant	
Rep	2	130.1029	65.0515	1.4245	0.2665	NS	
Trt	9	2905.1086	322.7898	7.0682	0.0002	**	
Error	18	822.0170	45.6676	.	.	-	
Corrected Total	29	3857.2285	.	.	.	-	
** - Significant at 1%, * - Significant  at 5%, NS - Non Significant	


Analysis Performed at IASRI Server


Block Design Experiment at NA For NA
ANOVA Analysis - Dependent Variable is _1s0

Source	DF	Type III SS	Mean Square	F Value	Pr > F	Significant	
Rep	2	224.3797	112.1899	4.5469	0.0252	*	
Trt	9	935.0741	103.8971	4.2108	0.0046	**	
Error	18	444.1340	24.6741	.	.	-	
Corrected Total	29	1603.5879	.	.	.	-	
** - Significant at 1%, * - Significant  at 5%, NS - Non Significant	


Analysis Performed at IASRI Server


Block Design Experiment at NA For NA
ANOVA Analysis - Dependent Variable is _1s1

Source	DF	Type III SS	Mean Square	F Value	Pr > F	Significant	
Rep	2	126.7208	63.3604	3.3984	0.0560	NS	
Trt	9	407.2612	45.2512	2.4271	0.0522	NS	
Error	18	335.5994	18.6444	.	.	-	
Corrected Total	29	869.5814	.	.	.	-	
** - Significant at 1%, * - Significant  at 5%, NS - Non Significant	


Analysis Performed at IASRI Server


Block Design Experiment at NA For NA
ANOVA Analysis - Dependent Variable is _1s2

Source	DF	Type III SS	Mean Square	F Value	Pr > F	Significant	
Rep	2	99.3459	49.6729	3.7191	0.0445	*	
Trt	9	746.7642	82.9738	6.2123	0.0005	**	
Error	18	240.4142	13.3563	.	.	-	
Corrected Total	29	1086.5243	.	.	.	-	
** - Significant at 1%, * - Significant  at 5%, NS - Non Significant	


Analysis Performed at IASRI Server


Block Design Experiment at NA For NA
ANOVA Analysis - Dependent Variable is _2s0

Source	DF	Type III SS	Mean Square	F Value	Pr > F	Significant	
Rep	2	392.6922	196.3461	4.4030	0.0278	*	
Trt	9	1316.2557	146.2506	3.2796	0.0153	*	
Error	18	802.6861	44.5937	.	.	-	
Corrected Total	29	2511.6341	.	.	.	-	
** - Significant at 1%, * - Significant  at 5%, NS - Non Significant	


Analysis Performed at IASRI Server


Block Design Experiment at NA For NA
ANOVA Analysis - Dependent Variable is _2s1

Source	DF	Type III SS	Mean Square	F Value	Pr > F	Significant	
Rep	2	21.0061	10.5031	0.6206	0.5487	NS	
Trt	9	342.4654	38.0517	2.2483	0.0686	NS	
Error	18	304.6453	16.9247	.	.	-	
Corrected Total	29	668.1169	.	.	.	-	
** - Significant at 1%, * - Significant  at 5%, NS - Non Significant	


Analysis Performed at IASRI Server


Block Design Experiment at NA For NA
ANOVA Analysis - Dependent Variable is _2s2

Source	DF	Type III SS	Mean Square	F Value	Pr > F	Significant	
Rep	2	183.1215	91.5608	4.1352	0.0333	*	
Trt	9	854.7976	94.9775	4.2895	0.0042	**	
Error	18	398.5564	22.1420	.	.	-	
Corrected Total	29	1436.4755	.	.	.	-	
** - Significant at 1%, * - Significant  at 5%, NS - Non Significant	


Analysis Performed at IASRI Server


Block Design Experiment at NA For NA
ANOVA Analysis - Dependent Variable is _2s3

Source	DF	Type III SS	Mean Square	F Value	Pr > F	Significant	
Rep	2	560.2465	280.1232	7.0544	0.0055	**	
Trt	9	2379.8551	264.4283	6.6592	0.0003	**	
Error	18	714.7597	39.7089	.	.	-	
Corrected Total	29	3654.8613	.	.	.	-	
** - Significant at 1%, * - Significant  at 5%, NS - Non Significant	


Analysis Performed at IASRI Server


Block Design Experiment at NA For NA
ANOVA Analysis - Dependent Variable is _3s0

Source	DF	Type III SS	Mean Square	F Value	Pr > F	Significant	
Rep	2	812.3478	406.1739	5.9640	0.0103	*	
Trt	9	601.5761	66.8418	0.9815	0.4868	NS	
Error	18	1225.8721	68.1040	.	.	-	
Corrected Total	29	2639.7960	.	.	.	-	
** - Significant at 1%, * - Significant  at 5%, NS - Non Significant	


Analysis Performed at IASRI Server


Block Design Experiment at NA For NA
ANOVA Analysis - Dependent Variable is _3s1

Source	DF	Type III SS	Mean Square	F Value	Pr > F	Significant	
Rep	2	22.2587	11.1294	0.6321	0.5429	NS	
Trt	9	665.5648	73.9516	4.2001	0.0046	**	
Error	18	316.9289	17.6072	.	.	-	
Corrected Total	29	1004.7524	.	.	.	-	
** - Significant at 1%, * - Significant  at 5%, NS - Non Significant	


Analysis Performed at IASRI Server


Block Design Experiment at NA For NA
ANOVA Analysis - Dependent Variable is _3s2

Source	DF	Type III SS	Mean Square	F Value	Pr > F	Significant	
Rep	2	784.8715	392.4358	8.2780	0.0028	**	
Trt	9	1199.0028	133.2225	2.8102	0.0297	*	
Error	18	853.3248	47.4069	.	.	-	
Corrected Total	29	2837.1991	.	.	.	-	
** - Significant at 1%, * - Significant  at 5%, NS - Non Significant	


Analysis Performed at IASRI Server


Block Design Experiment at NA For NA
ANOVA Analysis - Dependent Variable is _3s3

Source	DF	Type III SS	Mean Square	F Value	Pr > F	Significant	
Rep	2	1506.6093	753.3047	8.6934	0.0023	**	
Trt	9	703.1605	78.1289	0.9016	0.5438	NS	
Error	18	1559.7497	86.6528	.	.	-	
Corrected Total	29	3769.5196	.	.	.	-	
** - Significant at 1%, * - Significant  at 5%, NS - Non Significant	


Analysis Performed at IASRI Server


Block Design Experiment at NA For NA
ANOVA Analysis - Dependent Variable is _3s4

Source	DF	Type III SS	Mean Square	F Value	Pr > F	Significant	
Rep	2	115.7850	57.8925	2.2261	0.1368	NS	
Trt	9	1048.6247	116.5139	4.4803	0.0033	**	
Error	18	468.1092	26.0061	.	.	-	
Corrected Total	29	1632.5189	.	.	.	-	
** - Significant at 1%, * - Significant  at 5%, NS - Non Significant	


Analysis Performed at IASRI Server


Block Design Experiment at NA For NA
ANOVA Analysis - Dependent Variable is _4s0

Source	DF	Type III SS	Mean Square	F Value	Pr > F	Significant	
Rep	2	706.6194	353.3097	4.5251	0.0256	*	
Trt	9	820.8104	91.2012	1.1681	0.3704	NS	
Error	18	1405.4098	78.0783	.	.	-	
Corrected Total	29	2932.8396	.	.	.	-	
** - Significant at 1%, * - Significant  at 5%, NS - Non Significant	


Analysis Performed at IASRI Server


Block Design Experiment at NA For NA
ANOVA Analysis - Dependent Variable is _4s1

Source	DF	Type III SS	Mean Square	F Value	Pr > F	Significant	
Rep	2	58.5537	29.2768	0.7599	0.4821	NS	
Trt	9	531.9389	59.1043	1.5341	0.2099	NS	
Error	18	693.4757	38.5264	.	.	-	
Corrected Total	29	1283.9683	.	.	.	-	
** - Significant at 1%, * - Significant  at 5%, NS - Non Significant	


Analysis Performed at IASRI Server


Block Design Experiment at NA For NA
ANOVA Analysis - Dependent Variable is _4s2

Source	DF	Type III SS	Mean Square	F Value	Pr > F	Significant	
Rep	2	0.2389	0.1194	0.0023	0.9977	NS	
Trt	9	683.0467	75.8941	1.4616	0.2353	NS	
Error	18	934.6702	51.9261	.	.	-	
Corrected Total	29	1617.9558	.	.	.	-	
** - Significant at 1%, * - Significant  at 5%, NS - Non Significant	


Analysis Performed at IASRI Server


Block Design Experiment at NA For NA
ANOVA Analysis - Dependent Variable is _4s3

Source	DF	Type III SS	Mean Square	F Value	Pr > F	Significant	
Rep	2	43.5923	21.7962	0.9635	0.4004	NS	
Trt	9	484.3596	53.8177	2.3791	0.0562	NS	
Error	18	407.1724	22.6207	.	.	-	
Corrected Total	29	935.1243	.	.	.	-	
** - Significant at 1%, * - Significant  at 5%, NS - Non Significant	


Analysis Performed at IASRI Server


Block Design Experiment at NA For NA
ANOVA Analysis - Dependent Variable is _4s4

Source	DF	Type III SS	Mean Square	F Value	Pr > F	Significant	
Rep	2	356.2581	178.1291	3.9508	0.0378	*	
Trt	9	1218.0804	135.3423	3.0018	0.0225	*	
Error	18	811.5572	45.0865	.	.	-	
Corrected Total	29	2385.8958	.	.	.	-	
** - Significant at 1%, * - Significant  at 5%, NS - Non Significant	


Analysis Performed at IASRI Server


Block Design Experiment at NA For NA
ANOVA Analysis - Dependent Variable is _4s5

Source	DF	Type III SS	Mean Square	F Value	Pr > F	Significant	
Rep	2	8.4073	4.2036	0.3227	0.7283	NS	
Trt	9	747.0003	83.0000	6.3720	0.0004	**	
Error	18	234.4639	13.0258	.	.	-	
Corrected Total	29	989.8715	.	.	.	-	
** - Significant at 1%, * - Significant  at 5%, NS - Non Significant	


Analysis Performed at IASRI Server


Block Design Experiment at NA For NA
ANOVA Analysis - Dependent Variable is _5s0

Source	DF	Type III SS	Mean Square	F Value	Pr > F	Significant	
Rep	2	706.6194	353.3097	4.5251	0.0256	*	
Trt	9	820.8104	91.2012	1.1681	0.3704	NS	
Error	18	1405.4098	78.0783	.	.	-	
Corrected Total	29	2932.8396	.	.	.	-	
** - Significant at 1%, * - Significant  at 5%, NS - Non Significant	


Analysis Performed at IASRI Server


Block Design Experiment at NA For NA
ANOVA Analysis - Dependent Variable is _5s1

Source	DF	Type III SS	Mean Square	F Value	Pr > F	Significant	
Rep	2	58.5537	29.2768	0.7599	0.4821	NS	
Trt	9	531.9389	59.1043	1.5341	0.2099	NS	
Error	18	693.4757	38.5264	.	.	-	
Corrected Total	29	1283.9683	.	.	.	-	
** - Significant at 1%, * - Significant  at 5%, NS - Non Significant	


Analysis Performed at IASRI Server


Block Design Experiment at NA For NA
ANOVA Analysis - Dependent Variable is _5s2

Source	DF	Type III SS	Mean Square	F Value	Pr > F	Significant	
Rep	2	11.1301	5.5650	0.1304	0.8785	NS	
Trt	9	383.9882	42.6654	1.0000	0.4742	NS	
Error	18	767.9763	42.6654	.	.	-	
Corrected Total	29	1163.0946	.	.	.	-	
** - Significant at 1%, * - Significant  at 5%, NS - Non Significant	


Analysis Performed at IASRI Server


Block Design Experiment at NA For NA
ANOVA Analysis - Dependent Variable is _5s3

Source	DF	Type III SS	Mean Square	F Value	Pr > F	Significant	
Rep	2	75.4018	37.7009	1.7347	0.2047	NS	
Trt	9	932.3812	103.5979	4.7667	0.0024	**	
Error	18	391.2098	21.7339	.	.	-	
Corrected Total	29	1398.9929	.	.	.	-	
** - Significant at 1%, * - Significant  at 5%, NS - Non Significant	


Analysis Performed at IASRI Server


Block Design Experiment at NA For NA
ANOVA Analysis - Dependent Variable is _5s4

Source	DF	Type III SS	Mean Square	F Value	Pr > F	Significant	
Rep	2	375.8912	187.9456	3.2140	0.0640	NS	
Trt	9	1065.2849	118.3650	2.0241	0.0970	NS	
Error	18	1052.5848	58.4769	.	.	-	
Corrected Total	29	2493.7609	.	.	.	-	
** - Significant at 1%, * - Significant  at 5%, NS - Non Significant	


Analysis Performed at IASRI Server


Block Design Experiment at NA For NA
ANOVA Analysis - Dependent Variable is _5s5

Source	DF	Type III SS	Mean Square	F Value	Pr > F	Significant	
Rep	2	1.2624	0.6312	0.0486	0.9527	NS	
Trt	9	1291.8631	143.5403	11.0433	<.0001	**	
Error	18	233.9634	12.9980	.	.	-	
Corrected Total	29	1527.0889	.	.	.	-	
** - Significant at 1%, * - Significant  at 5%, NS - Non Significant	


Analysis Performed at IASRI Server


Block Design Experiment at NA For NA
ANOVA Analysis - Dependent Variable is _6s5

Source	DF	Type III SS	Mean Square	F Value	Pr > F	Significant	
Rep	2	165.7665	82.8833	2.4745	0.1124	NS	
Trt	9	1273.4207	141.4912	4.2243	0.0045	**	
Error	18	602.9096	33.4950	.	.	-	
Corrected Total	29	2042.0969	.	.	.	-	
** - Significant at 1%, * - Significant  at 5%, NS - Non Significant	


Analysis Performed at IASRI Server


Block Design Experiment at NA For NA
ANOVA Analysis - Dependent Variable is _7s5

Source	DF	Type III SS	Mean Square	F Value	Pr > F	Significant	
Rep	2	69.4996	34.7498	0.8634	0.4385	NS	
Trt	9	1484.3903	164.9323	4.0978	0.0053	**	
Error	18	724.4804	40.2489	.	.	-	
Corrected Total	29	2278.3703	.	.	.	-	
** - Significant at 1%, * - Significant  at 5%, NS - Non Significant	


Analysis Performed at IASRI Server


Block Design Experiment at NA For NA
ANOVA Analysis - Dependent Variable is _8s5

Source	DF	Type III SS	Mean Square	F Value	Pr > F	Significant	
Rep	2	85.5898	42.7949	1.0874	0.3582	NS	
Trt	9	1601.3892	177.9321	4.5212	0.0032	**	
Error	18	708.3902	39.3550	.	.	-	
Corrected Total	29	2395.3692	.	.	.	-	
** - Significant at 1%, * - Significant  at 5%, NS - Non Significant	


Analysis Performed at IASRI Server


Block Design Experiment at NA For NA
ANOVA Analysis - Dependent Variable is _9s5

Source	DF	Type III SS	Mean Square	F Value	Pr > F	Significant	
Rep	2	133.7033	66.8516	1.4816	0.2537	NS	
Trt	9	2490.7257	276.7473	6.1336	0.0006	**	
Error	18	812.1592	45.1200	.	.	-	
Corrected Total	29	3436.5881	.	.	.	-	
** - Significant at 1%, * - Significant  at 5%, NS - Non Significant	


Analysis Performed at IASRI Server


Block Design Experiment at NA For NA
Treatment Mean Table

Treatment Name	Treatment Description	Mgt	Sgi	Svi__1	Var35	_10s5	_1s0	_1s1	_1s2	_2s0	_2s1	
1		16.87	4.52	2.13	6.18	0.00	50.79	33.21	18.05	41.13	29.93	
2		16.39	4.88	2.30	7.09	12.29	50.96	34.02	16.60	37.87	26.07	
3		15.97	5.36	3.01	9.89	32.02	47.96	34.04	20.76	39.01	25.00	
4		17.26	4.87	2.31	7.42	8.61	47.91	35.22	19.50	39.04	32.02	
5		16.44	5.67	2.52	8.68	14.76	48.08	33.73	21.34	36.61	26.57	
6		13.10	6.05	2.18	6.18	0.00	42.12	33.16	29.93	33.16	26.45	
7		17.65	4.31	1.96	5.98	11.90	52.74	27.71	22.79	44.04	34.18	
8		13.85	5.60	2.51	10.06	28.67	45.97	29.93	28.67	35.17	28.86	
9		14.00	6.55	2.52	9.30	12.29	37.26	37.26	31.07	23.86	35.25	
10		13.99	7.00	2.83	9.34	12.92	35.25	42.13	27.71	22.79	27.71	
General Mean		15.55	5.48	2.43	8.01	13.35	45.90	34.04	23.64	35.27	29.20	
p-Value		<.0001	0.0661	0.4560	0.1421	0.0002	0.0046	0.0522	0.0005	0.0153	0.0686	
CV(%)		4.35	18.33	22.53	26.24	50.64	10.82	12.68	15.46	18.93	14.09	
SE(d)		0.553	0.820	0.446	1.716	5.518	4.056	3.526	2.984	5.452	3.359	
LSD at 5%		1.1618	NS	NS	NS	11.592	8.5209	NS	6.2691	11.455	NS	

_2s2	_2s3	_3s0	_3s1	_3s2	_3s3	_3s4	_4s0	_4s1	_4s2	_4s3	_4s4	_4s5	_5s0	
24.05	22.60	32.30	16.60	24.23	37.60	12.92	31.31	14.76	12.92	31.07	35.00	-0.00	31.31	
33.16	19.31	30.19	14.76	15.00	40.81	20.76	30.19	8.61	8.61	35.01	35.17	4.31	30.19	
32.76	16.45	26.41	12.92	18.61	38.10	25.25	22.10	14.76	4.31	23.86	46.92	16.60	22.10	
28.24	16.60	31.45	10.45	18.61	44.43	12.92	21.52	22.29	4.31	27.22	42.09	0.00	21.52	
31.75	18.86	24.98	12.92	22.54	41.53	20.76	19.50	14.31	10.45	23.74	48.93	0.00	19.50	
35.25	24.05	27.71	22.60	8.61	36.24	29.93	27.71	21.34	4.31	33.16	35.25	-0.00	27.71	
25.31	-0.00	32.09	23.86	21.34	31.07	22.60	32.09	19.89	16.60	31.07	29.93	0.00	32.09	
27.71	27.60	33.16	19.89	16.60	41.15	14.76	33.16	16.60	10.45	22.60	40.20	4.31	33.16	
43.09	8.61	21.34	24.05	4.31	47.88	21.34	21.34	21.34	4.31	29.93	43.09	0.00	21.34	
35.17	33.16	19.89	14.76	8.61	46.95	29.93	19.89	12.92	-0.00	29.93	49.80	0.00	19.89	
31.65	18.72	27.95	17.28	15.85	40.58	21.12	25.88	16.68	7.63	28.76	40.64	2.52	25.88	
0.0042	0.0003	0.4868	0.0046	0.0297	0.5438	0.0033	0.3704	0.2099	0.2353	0.0562	0.0225	0.0004	0.3704	
14.87	33.66	29.52	24.28	43.45	22.94	24.15	34.14	37.21	94.49	16.54	16.52	143.16	34.14	
3.842	5.145	6.738	3.426	5.622	7.600	4.164	7.215	5.068	5.884	3.883	5.482	2.947	7.215	
8.0718	10.81	NS	7.198	11.811	NS	8.7479	NS	NS	NS	NS	11.518	6.1911	NS	

_5s1	_5s2	_5s3	_5s4	_5s5	_6s5	_7s5	_8s5	_9s5	
14.76	8.61	28.86	38.05	-0.00	0.00	-0.00	0.00	-0.00	
8.61	8.61	29.74	40.11	8.61	8.61	10.45	10.45	12.29	
14.76	4.31	21.14	48.93	21.34	22.60	24.05	25.31	31.00	
22.29	0.00	20.76	48.90	0.00	0.00	4.31	4.31	8.61	
14.31	8.61	21.34	52.11	0.00	4.31	8.61	8.61	14.76	
21.34	4.31	28.86	39.21	0.00	0.00	0.00	-0.00	-0.00	
19.89	8.61	27.71	36.24	0.00	4.31	8.61	8.61	11.90	
16.60	0.00	10.45	48.84	4.31	10.45	18.05	18.05	25.00	
21.34	4.31	26.45	45.96	0.00	8.61	8.61	8.61	12.29	
12.92	0.00	25.31	53.73	0.00	4.31	8.61	8.61	12.92	
16.68	4.74	24.06	45.21	3.43	6.32	9.13	9.26	12.88	
0.2099	0.4742	0.0024	0.0970	<.0001	0.0045	0.0053	0.0032	0.0006	
37.21	137.87	19.38	16.92	105.24	91.58	69.48	67.77	52.16	
5.068	5.333	3.806	6.244	2.944	4.725	5.180	5.122	5.485	
NS	NS	7.9971	NS	6.1845	9.9278	10.883	10.761	11.523	


Means with atleast one letter common are not statistically significant using
Fisher's Least Significant Difference


Analysis Performed at IASRI Server


Block Design Experiment at NA For NA
Treatment Details Table

Obs	Treatment Name	Treatment Details	
1	1		
2	2		
3	3		
4	4		
5	5		
6	6		
7	7		
8	8		
9	9		
10	10		


Analysis Performed at IASRI Server
